# Evolution Stings: The Origin and Diversification of Scorpion Toxin Peptide Scaffolds

**DOI:** 10.3390/toxins5122456

**Published:** 2013-12-13

**Authors:** Kartik Sunagar, Eivind A. B. Undheim, Angelo H. C. Chan, Ivan Koludarov, Sergio A. Muñoz-Gómez, Agostinho Antunes, Bryan G. Fry

**Affiliations:** CIMAR/CIIMAR, Centro Interdisciplinar de Investigação Marinha e Ambiental, Universidade do Porto, Rua dos Bragas, 177, 4050-123 Porto, Portugal; E-Mails: anaturalist@gmail.com (K.S.); aantunes777@gmail.com (A.A.); Departamento de Biologia, Faculdade de Ciências, Universidade do Porto, Rua do Campo Alegre, 4169-007, Porto, Portugal; Venom Evolution Lab, School of Biological Sciences, The University of Queensland, St. Lucia, Queensland 4072, Australia; E-Mails: eivindandreas@gmail.com (E.A.B.U.); angelo.hoi.chung.chan@gmail.com (A.H.C.C.); jcoludar@gmail.com (I.K.); Institute for Molecular Bioscience, The University of Queensland, St. Lucia, Queensland 4072, Australia; Department of Biochemistry and Molecular Biology, Centre for Comparative Genomics and Evolutionary Bioinformatics, Dalhousie University, Halifax, Nova Scotia, Canada; E-Mail: sergio.munoz@dal.ca

**Keywords:** adaptive evolution, scorpion venom arsenal, scorpion toxin scaffolds

## Abstract

The episodic nature of natural selection and the accumulation of extreme sequence divergence in venom-encoding genes over long periods of evolutionary time can obscure the signature of positive Darwinian selection. Recognition of the true biocomplexity is further hampered by the limited taxon selection, with easy to obtain or medically important species typically being the subject of intense venom research, relative to the actual taxonomical diversity in nature. This holds true for scorpions, which are one of the most ancient terrestrial venomous animal lineages. The family Buthidae that includes all the medically significant species has been intensely investigated around the globe, while almost completely ignoring the remaining non-buthid families. Australian scorpion lineages, for instance, have been completely neglected, with only a single scorpion species (*Urodacus yaschenkoi*) having its venom transcriptome sequenced. Hence, the lack of venom composition and toxin sequence information from an entire continent’s worth of scorpions has impeded our understanding of the molecular evolution of scorpion venom. The molecular origin, phylogenetic relationships and evolutionary histories of most scorpion toxin scaffolds remain enigmatic. In this study, we have sequenced venom gland transcriptomes of a wide taxonomical diversity of scorpions from Australia, including buthid and non-buthid representatives. Using state-of-art molecular evolutionary analyses, we show that a majority of CSα/β toxin scaffolds have experienced episodic influence of positive selection, while most non-CSα/β linear toxins evolve under the extreme influence of negative selection. For the first time, we have unraveled the molecular origin of the major scorpion toxin scaffolds, such as scorpion venom single von Willebrand factor C-domain peptides (SV-SVC), inhibitor cystine knot (ICK), disulphide-directed beta-hairpin (DDH), bradykinin potentiating peptides (BPP), linear non-disulphide bridged peptides and antimicrobial peptides (AMP). We have thus demonstrated that even neglected lineages of scorpions are a rich pool of novel biochemical components, which have evolved over millions of years to target specific ion channels in prey animals, and as a result, possess tremendous implications in therapeutics.

## 1. Introduction

The scientific consensus is that venom-encoding genes undergo dynamic molecular evolution, predominantly as a result of variations in prey preference and/or predatory strategy employed [1,2,3,4,5,6,7,8,9,10,11,12]. The underlying molecular diversity of venom however, could be obscured by the episodic nature of selection on genes that encode them and the extreme sequence divergence that occurs over long periods of evolutionary time. This is particularly true for venom-encoding genes in some of the oldest venomous lineages, such as cnidarians, centipedes, scorpions, spiders, coleoids, *etc.* In addition, the full recognition of natural complexity is hampered by the limited taxon selection in biodiscovery oriented research, with easy to obtain or medically important species typically being the subject to a disproportionate level of research relative to the true taxonomical diversity [13].

Scorpions are an ancient and species rich terrestrial venomous lineage. Buthidae is not only the largest family among scorpion lineages, but is also the one that includes all the medically significant species. As apparent from the extreme potency of their venom and the relatively smaller pincers in comparison to a rather large stinger, which is particularly pronounced in the fat-tailed scorpions genera (*Androctonus* and *Parabuthus*), members of this family heavily rely on their venom arsenal for predation. This is in contrast with the non-buthid families that use a combined arsenal of strong pincers and envenoming for prey-subjugation, with the pincers as the primary weapon.

Over the course of 400+ million years of evolutionary time [14], scorpions have evolved venoms that exert toxic activities on a wide range of biological targets. The toxin diversity has been potentiated by the slow migration rates and divergent population structures combined with their long geological history [15,16,17,18,19]. In contrast to some snake venoms which are rich in enzymatic toxins, scorpion venoms are dominated by peptide toxins (Table 1). Among several toxin types present in scorpion venoms (see [13] for a review), the CSα/β scaffold (cysteine-stabilised α/β) are particularly complex. Scorpion CSα/β toxins include sodium ion channel (Na_V_) modulators (NaScTx or Na_V_-CSα/β; subtypes: α-toxins (site-3 binding) and β-toxins (site-4 binding)), atypical Na_V_-CSα/β (birtoxin and similar toxins including the so-called ‘lipolytic toxins’), potassium ion channel (Kv) targeting toxins (KTx) (short and long subtypes) and the chloride toxin family [20,21,22,23,24].

**Table 1:** Major groups of scorpion venom peptides.

**Toxin Type**	**Generalised Bioactivity**	**Representative Key References**
**Cytotoxin “bradykinin potentiating peptide family”**	Potent cytotoxins leading to cell haemolysis and death. Documented antimicrobial activity is a side effect due to generalised cell-killing. C-terminal coil region contributes additional activity of bradykinin potentiation.	[25,26,27]
**Cytotoxin “NDBP 5 linear peptide family”**	Potent cytotoxins leading to cell haemolysis and death. Documented antimicrobial activity is a side effect due to generalised cell-killing.	[28,29,30,31]
**Cytoxin–“Short cationic antimicrobial peptide family”**	Potent cytotoxins leading to cell haemolysis and death. Documented antimicrobial activity is a side effect due to generalised cell-killing.	[30,32,33,34]
**Anionic**	uncharacterised	[27,32,35,36,37,38]
**Glycine-rich**	uncharacterised	[27]
**CSαβ**		
***CS*αβ *‘alpha’***	Prevent inactivation by binding to sodium channel receptor site 3	[13,39,40,41,42,43,44,45]
***CS*αβ *‘beta’***	Promote activation by binding to sodium channel receptor site 4	
***CS*αβ *‘lipo’***	Lipolysis	
***CS*αβ *‘chlorotoxin’***	Chloride channel	
***CS*αβ *‘short-chain’***	Potassium channel	
***CS*αβ *‘long-chain’***	Potassium channel and antimicrobial	
***CS*αβ *‘scorpine’***	Potassium channel and antimicrobial	
**ICK/DDH**		
***SV-SVC***	Functionally uncharacterised	[27,46,47,48]
***ICK***	Strong agonist of ryanodine receptors (calcium release channels). Induces voltage- and concentration-dependent subconductance states in both skeletal (RYR1 and RYR3) and cardiac (RYR2) ryanodine receptors by binding to a single, cytosolically accessible site different from the ryanodine binding site. Enhances calcium release. A derivative (GU187948) inhibits Shaker K^+^ channels	[27,42,49,50,51,52,53,54,55,56,57,58,59,60,61]
***DDH***	Types such as P60252 from *Opistophthalmus carinatus*, P59868 from *Pandinus imperator* and B8QG00 from *Hadrurus gertschi* potently and reversibly modify channel gating behavior of the type 1 ryanodine receptor (RYR1) by inducing prominent subconductance behavior. Binds a different site as ryanodine. Others with different cysteine frameworks such as P0DJ08 *Liocheles waigiensis* are insect-selective toxins. Provokes a dose-dependent contractile paralysis in crickets and blowfly larvae, followed by death.	[48,62,63,64,65,66]

Despite this recognised complexity, the relative phylogenetic relationships and the molecular evolutionary histories of most scorpion toxin types remain enigmatic. Similarly, while scorpions have globally been the subject of intensive research, the species in Australia have been largely neglected by toxicological research. Only one scorpion species (*Urodacus yaschenkoi*) from Australia has had its venom transcriptome sequenced to date [67]. Only few other studies have examined isolated peptides: two nearly identical DDH (disulphide-directed β-hairpin) peptides from two very closely related scorpion species (*Liocheles australasiae* and *L. waigiensis* [62,63,64]; and a third examining a peptide related to the SVC arthropod peptides [46]. Such a scarcity of knowledge from an entire continent’s worth of scorpions has hampered our understanding of scorpion venom peptide molecular evolution. Thus, in this study we selected representatives of the taxonomical diversity of buthid and non-buthid species of Australian scorpions in order to examine the molecular complexity through transcriptome sequencing. These new sequences enabled us to robustly reconstruct the complex molecular evolutionary histories of various scorpion peptide toxin types and evaluate the influence of natural selection on their evolution.

## 2. Results and Discussion

### 2.1. Transcriptomics of Scorpion Venom-Glands Highlights the True Diversity of Scorpion Venom-Arsenal

We obtained over 66,000 reads per transcriptome with an average length of 362 bases. Automatic assembly provided an average of 4,307 contigs per transcriptome with an average length of 574 bases. Assembly details are provided in Supplementary Table 1. BLAST searches revealed the transcriptomes to contain a myriad of peptides and proteins. Public access of the data can be found at the National Center for Biotechnology Information (NCBI) under Bioprojects: *Australobuthus xerlomnion* PRJNA201348; Cercophonius squama PRJNA201349; *Isometroides vescus* PRJNA201350; *Lychas buchari* PRJNA201351; *Urodacus manicatus* PRJNA201352. Sequenced analysed in this study have the Genbank accession numbers of: *Australobuthus xerlomnion* GALG01000001-GALG01000002; *Cercophonius squama* GALH01000001-GALH01000013; *Isometroides vescus* GALK01000001-GALK01000018; *Lychas buchari* GALL01000001-GALL01000032; *Urodacus manicatus* GALI01000001-GALI01000019.

For the purpose of this study we focused upon the wide diversity of peptide toxin types (Table 2) recovered in our libraries. We obtained novel sequences from three families of linear toxin peptides (cytotoxic, anionic and glycine-rich): (i) the first cytotoxin peptide sequences from members of the Bothriuridae family (3 from *Cercophonius squama*) and the Urodacidae family (5 from *Urodacus manicatus*); (ii) the first anionic peptide sequences from a member of the Urodacidae family (1 from *Urodacus. manicatus*), as well as additional isoforms from Bothriuridae (1 from *Cercophonius squama*) and Buthidae (3 from *Isometroides vescus* and two from *Lychas buchari*) which like other members of this peptide type, had extremely negative charges (pI 2); and (iii) the first glycine-rich peptide sequence from a member of the Urodacidae family (1 from *Urodacus manicatus*), which also represents the first sequences from a non-buthid scorpions, as well as additional Buthidae isoforms (4 from *Lychas buchari*). In addition, we were able to retrieve the first DDH sequences from a member of the Urodacidae family (3 from *Urodacus manicatus*), and the first SV-SVC sequences from members of the Bothriuridae family (2 from *Cercophonius squama*) in addition to Buthidae SV-SVC isoforms (3 from *Lychas buchari*). We also recovered an extensive array of CSα/β isoforms: 1 from *Australobuthus xerlomnion*; 6 from *Cercophonius squama*; 8 from *Isometroides vescus*; 16 from *Lychas buchari*; and 4 from *Urodacus manicatus*.

**Table 2:** Major groups of scorpion venom peptides recovered by transcriptome sequencing of Australian scorpions.

	Species	*Australobuthus xerlomnion*	*Cercophonius* *squama*	*Isometroides vescus*	*Lychas* *buchari*	*Urodacus manicatus*
**Toxin Type**						
**Cytotoxin “bradykinin potentiating peptide family”**				X	X	
**Cytotoxin “NDBP 5 linear peptide family”**			X			X
**Cytotoxin–“Short cationic antimicrobial peptide family**				X	X	
**Anionic**			X	X	X	X
**CS*αβ***						
***CSαβ ‘alpha’ Na_V_***						
***CSαβ-‘beta’ Na_V_***				X		
***CSαβ ‘lipo’ Na_V_***			X		X	X
***CSαβ ‘chlorotoxin’***						
***CSαβ-‘short-chain’ K_V_***				X	X	
***CSαβ-‘long-chain’ K_V_***		X	X	X	X	X
***CSαβ ‘scorpine’ K_V_***			X			X
**Glycine**					X	
**ICK/DDH**						
***ICK***			X			
***DDH***						X
***SV-SVC***					X	

### 2.2. Implications for the Origin and Evolution of Scorpion Toxin Scaffolds

Sequence alignments and Bayesian phylogenetic analyses were employed to reconstruct the complex molecular evolutionary histories of the different scorpion peptide toxins, which revealed several fascinating results regarding their origin and diversification.

#### 2.2.1. Apotypic (Derived) Na_V_-CSα/β Scaffolds

**Figure 1:**
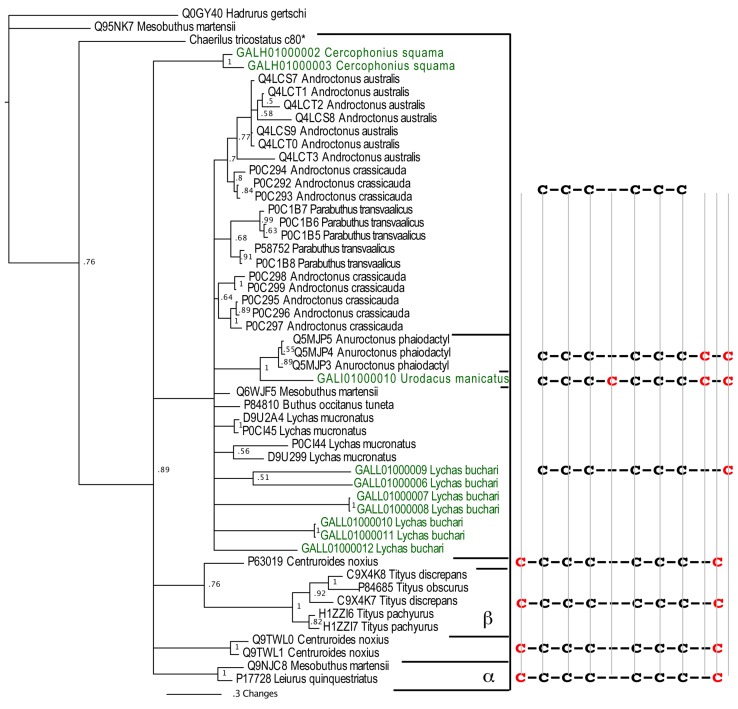
Bayesian phylogenetic reconstruction of the Na_V_-CSα/β clade**.** Outgroups were the K_V_-CSα/β Q0GY40 *Hadrurus gertschi* and Q95NK7 *Mesobuthus martensi*. *Chaerilus tricostatus contig sequence is from [71].

Consistent with the scorpion organismal evolutionary history, phylogenetic analyses in this study revealed that some non-buthid three-disulphide bond Na_V_-CSα/β sequences were plesiotypic relative to buthid Na_V_-CSα/β (Figure 1). However, reflective of the early derivation of NaV-CSα/β within scorpion venom prior to the splitting off of the Buthidae family, the 3 disulphide bond containing NaV-CSα/β were not reciprocally monophyletic between non-buthid and buthid species. As scorpion CSα/β peptides evolved from a six cysteine defensin peptide [68,39], this is consistent with our phylogenetic results that the 3 disulphide bond NaV-CSα/β are plesiotypic to the 4 disulphide bond containing α- and β-NaV-CSα/β toxins. Since the three disulphide bonded plesiotypic-Na_V_-CSα/β and the four disulphide bonded apotypic β-Na_V_-CSα/β toxins share the site-4 activity [69], this indicates that this is the plesiotypic functional activity of the Na_V_-CSα/β, with our phylogenetic results thus consistent with previously proposed functional evolution theories in this regard [39,70]. Thus the site-3 activity of the the α-Na_V_-CSα/β (which are stabilised by four disulphide bonds like the β-Na_V_-CSα/β) is functionally apotypic. 

Within the apotypic “lipolytic” group, the novel non-buthid sequences of this type rendered the buthid sequences non-monophyletic, thus indicating that this apotyposis within the Na_V_-CSα/β is early evolving prior to the splitting off of buthid scorpions. The lipolytic have a newly evolved 7th cysteine (located in the C-terminal region), thus giving an odd-number of cysteines and consequently promoting dimerization [72]. The non-buthid sequences within the lipolytic clade *Anuroctonus phaiodactyl* (Q5MJP3, Q5MJP4 and Q5MJP5) have a newly evolved 8th cysteine located in the C-terminal region and thus have an even number of cysteines (Figure 1). In the related non-buthid sequence form *Urodacus manicatus* (CSα/β-Uro-1), which shares this new cysteine with *A. phaiodactyl* sequences, an additional cysteine has been evolved, while a plesiotypic cysteine has been deleted. Thus the odd number of cysteines in this toxin type could consequently promote unique dimerization combinations. In contrast, the common ancestor of the α-Na_V_-CSα/β and β-Na_V_-CSα/β forms evolved two new cysteines with one in the C-terminal region and one located in the N-terminal region (Figure 1). Despite having evolved a novel cysteine residue in the C-terminal region, the lipolytic Na_V_-CSα/β and the α/β-Na_V_-CSα/β clades lack sequence similarity and form reciprocally non-monophyletic clades, suggesting that the two toxin types have independently evolved the additional cysteine residue (homoplasy).

#### 2.2.2. SV-SVCs: Putative Plesiotypic ICK-DDH Scaffold

**Figure 2:**
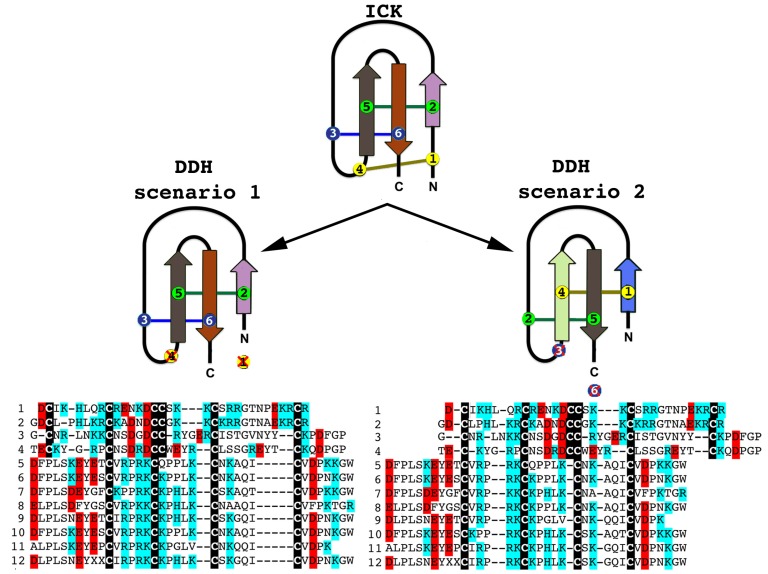
The two alternate scenarios of the cysteine relationships between DDH and ICK peptides. Sequences presented: 1. B8QG00 *Hadrurus gertschi*; 2. P59868 *Pandinus imperator*; 3. B8XH22 *Buthus occitanus israel*; 4. P0DJL0 *Isometrus maculatus*; 5. P0C5F2 *Liocheles australasiae*; 6. F8W670 *Liocheles australasiae*; 7. GALI01000016 *Urodacus manicatus*; 8. C5J894 *Opisthacanthus cayaporum*; 9. GALI01000015 *Urodacus manicatus*; 10. P0DJ08 *Liocheles waigiensis*; 11. SmpIT2 *Scorpio maurus palmatus* [66] and 12. GALI01000017 *Urodacus manicatus*. ICK connectivity schematic image adopted from [63]. Alignment scenario 1 is that proposed previously [63,64,65] while alignment scenario 2 is the alternative proposed in this study to better reflect charge molecule distribution.

**Figure 3:**
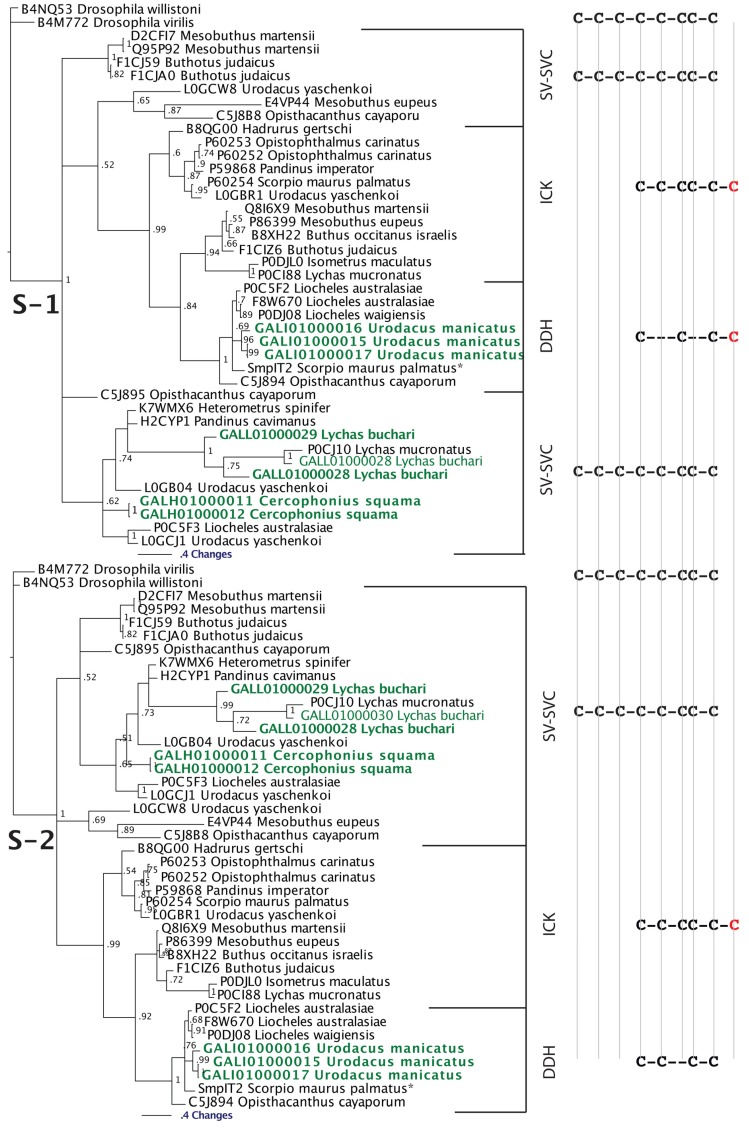
Bayesian phylogenetic reconstruction of the SV-SVC, ICK and DDH clade. Outgroups were the non-toxin SVC peptides B4M772 *Drosophila virilis* and B4NQ53 *Drosophila willistoni*. * SmpIT2 *Scorpio maurus palmatus* is from [66]. Alignment scenario 1 is that proposed previously [63,64,65] while alignment scenario 2 is the alternative proposed in this study to better reflect charge molecule distribution.

Although there have been suggestions in the past that the three-disulphide bond ICK fold has originated from the simpler, two-disulphide bond DDH scaffolds [63,73], phylogenetic investigations to unravel the precise evolutionary origin of ICK and DDH scaffolds remain unattempted to date. Based upon our analyses (Figure 2 and Figure 3), we suggest that the DDH are ICK derivatives. DDH and ICK differ by the number of cysteines, and thus disulphide bonds, with DDH having four cysteines and two disulphide bonds and ICK having six cysteines and three disulphide bonds. 

Of the shared cysteines, it has been previously proposed that they differ by the relative presence of the first and fourth of the ICK cysteines [63,64,65] (scenario 1 in Figure 2 and Figure 3). However, we propose an alternate scenario that the DDH and ICK differ by the relative presence of the third and sixth ICK cysteine (scenario 2 in Figure 2 and Figure 3). This scenario is more consistent in regards to the relative presence of charged resides. It should be noted, that it does not affect the relative structure of DDH toxins, but merely proposes an alternate evolutionary history of the cysteines shared between the DDH and ICK in regards to which ICK cysteines were deleted in the derivation to the DDH form. 

Our Bayesian molecular phylogenetic reconstructions investigated the link between SV-SVC peptides (scorpion venom single von Willebrand factor C-domain peptides) and the DDH and ICK peptides. The results of our phylogenetic reconstructions and sequence alignments clearly highlight the evolutionary origin of DDH and ICK sequences from the SV-SVC peptides (Figure 2 and Figure 3). Regardless of the alignment variance (full alignments of the two alternative scenarios can be found in the supplementary material), the DDH come out as derivatives of the ICK and the DDH/ICK clade is nested within the SV-SVC clade. The SV-SVC as the plesiotypic state for this entire clade is supported by this cysteine framework being widely distributed within arthropods [47], in comparison to the conspicuous absence of likely ancestral non-toxin arthropod forms of the ICK or DDH motifs. Hence, we rooted our tree using related arthropod non-toxin sequences that have been shown to be homologues of the SV-SVC [47]. It is noteworthy that SV-SVC have been retrieved from both buthid and non-buthid scorpion lineages, which is consistent with our single early evolution hypothesis. Similarly the SV-SVC apotypic forms (ICK and DDH) have been retrieved from both buthid and non-buthid venoms, indicative of their derivation occurring before the buthid split at the base of the scorpion evolution tree. While the DDH peptides have been proposed to be the plesiotypic state, with the ICK as an apotypic state [63], our results instead indicate that the DDH are actually a highly apotypic condition, phylogenetically nested within the ICK peptides, with the SV-SVC being the plesiotypic state and, as would be expected, are non-monophyletic relative to the DDH/ICK (Figure 2). The differences in cysteine pattern can be interpreted as a stepwise loss due to domain-deletions (Figure 3), which is consistent with the mapping of the known activities. 

While the SV-SVC are functionally uncharacterised, despite accounting for the majority of the venom in some species [46], the ICK and DDH both target and activate the ryanodine receptor intracellular calcium release channel (RyR). Biochemical testing of a DDH toxin on mammalian receptors revealed that it was more potent in action than the ICK peptides [64]. In contrast, the only buthid ICK ever tested appeared to lack the ability to modulate RyR, although it could block certain K+ channels at high concentration [49]. While the effects upon insect receptors remains unknown, the higher degree of potency of the DDH peptides on mammalian receptors in comparison to the ICK peptides [64] is in agreement with our proposed evolutionary model, as selection pressure is likely to produce a more refined and potent form of a framework with a useful activity. However, the functional pattern is consistent with molecular evolution patterns seen in other venoms such as exendin peptides in *Heloderma* venoms, where selection pressure has resulted in more potent cardiotoxic forms with apotypic sequence motifs [74] and also in the elapid snake venom 3FTx (three finger toxin) where selection pressure has resulted in more potent alpha-neurotoxic forms which lack two of the plesiotypic cysteines [4].

#### 2.2.3. Putative Common Origin of Cytotoxic Peptides

**Figure 4:**
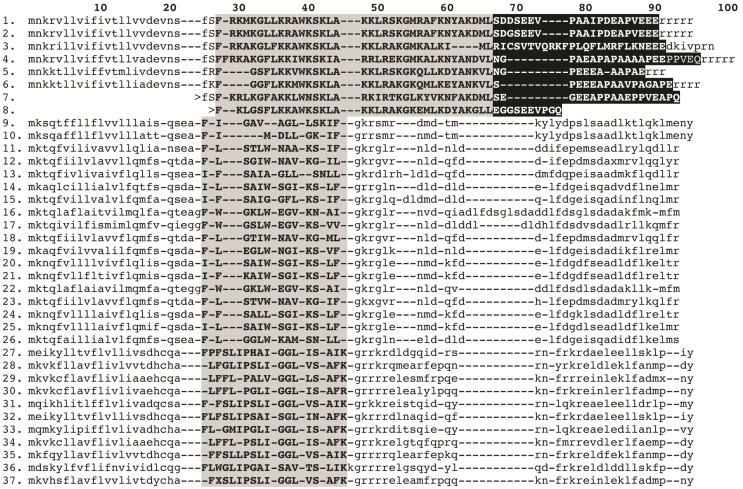
Sequence alignment of cytotoxic linear peptides: (1). GALK01000016 *Isometroides vescus*; (2). GALK01000016 *Isometroides vescus*; (3). GALL01000023 *Lychas buchari*; (4). D9U2B7 *Lychas mucronatus*; (5). Q9Y0X4 *Mesobuthus martensii*; (6). C9X4J0 *Tityus discrepans*; (7). P0CF38 *Isometrus maculatus*; (8). P83312 *Parabuthus schlechteri*; (9). Q9GQW4 *Mesobuthus martensii*; (10). B8XH50 *Buthus occitanus israelii*; (11). I0DEB4 *Vaejovis mexicanus smithii*; (12). GALH01000010 *Cercophonius squama*; (13). *P0C8W1 Hadrurus gertschi*; (14). C5J886 *Opisthacanthus cayaporum*; (15). P0DJ03 *Heterometrus petersii*; (16). L0GCV8 *Urodacus yaschenkoi*; (17). P0DJO3 *Scorpiops tibetanus*; (18). GALH01000009 *Cercophonius squama*; (19). GALI01000003 *Urodacus manicatus*; (20). GALI01000004 *Urodacus manicatus*; (21). GALI01000007 *Urodacus manicatus*; (22). GALI01000005 *Urodacus manicatus*; (23). GALH01000008 *Cercophonius squama*; (24). GALI01000006 *Urodacus manicatus*; (25). L0GCI6 *Urodacus yaschenkoi*; (26). H2CYR5 *Pandinus cavimanus*; (27). G8YYA6 *Androctonus amoreuxi*; (28). B9UIY3 *Lychas mucronatus*; (29). GALL01000021 *Lychas buchari*; (30). GALK01000015 *Isometroides vescus*; (31). Q5G8B3 *Tityus costatus*; (32). E4VP60 *Mesobuthus eupeus*; (33). Q5G8B5 *Tityus costatus*; (34). D9U2B8 *Lychas mucronatus*; (35). C7B247 *Isometrus maculatus*; (36). G1FE62 *Chaerilus tricostatus*; (37). GALL01000022 *Lychas buchari.* Signal peptide and C-terminal cleaved propeptides are shown in lowercase. BPP domain shown in black and the cytotoxic posttranslationally processed peptide is highlighted in gray. ‘>’ indicates incomplete sequence.

**Figure 5:**
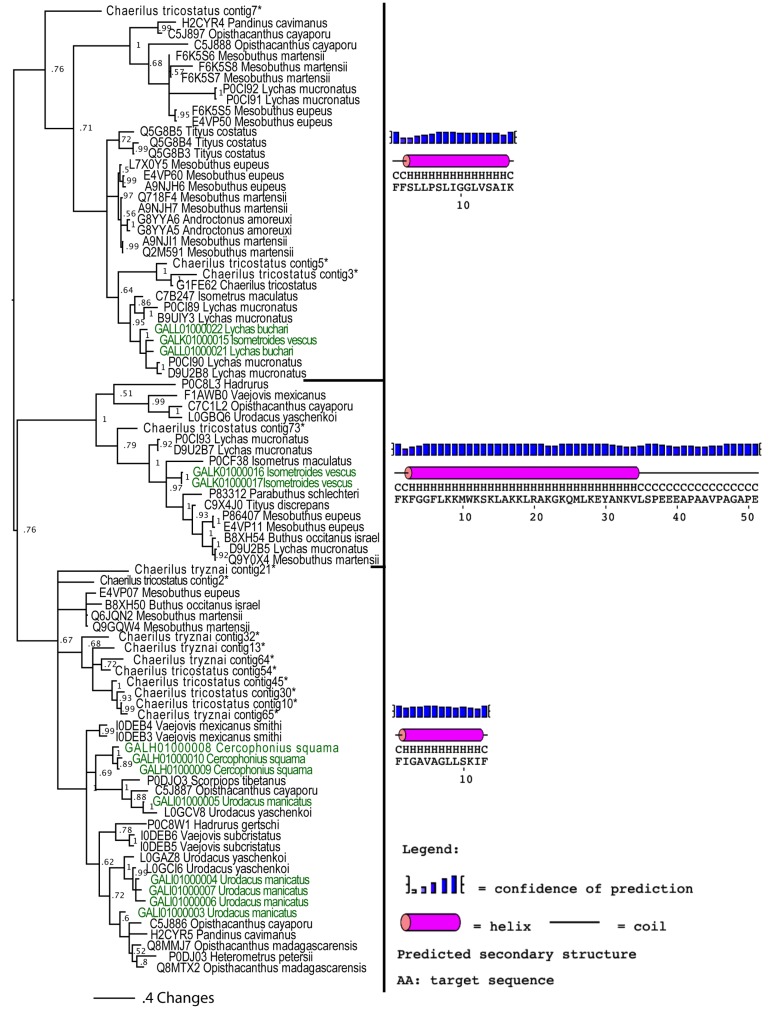
Mid-point rooted Bayesian phylogenetic reconstruction of the cytotoxic linear peptides. * *Chaerilus tricostatus* and *C. tryznai* contig sequences are from [71].

Our analyses suggested that the cytotoxic peptides (classified by the uniprot database into bradykinin potentiating peptides (BPP), linear non-disulphide bridged peptide (NDBP; referred to as linear henceforth) and short cationic antimicrobial peptide (AMP)) could have originated via a single early recruitment event. Despite the variations in sequences between the three clades (Figure 4), in all variants the posttranslationally liberated antimicrobial peptides formed well-developed alpha-helices (Figure 5). In addition to the conserved alpha-helix secondary structure [25,26,28], the cytolytic domain is characterised by extremely high pI values, with all forms having PI value that exceeds 8.5. PI value of this domain in the BPP clade approached 10.5. As these toxins have been shown to be lethal and synergistically enhance the excitatory effects of CSα/β ion channel-specific neurotoxins by interaction with the neuronal membranes [25,26,75,76,77,78,79], we consider any antimicrobial activity attributed to them in laboratory investigations (but without supporting natural history data) to be an incidental activity as a consequence of the powerful cytotoxicity, rather than an evolutionarily selected activity. This is in contrast to the alpha-helical frog antimicrobial peptides which play a protective role on the moist skin of frog, which is constantly targeted by microbial infections [80]. The cytolytic domain in BPP-type is followed by a highly variable C-terminal region random-coil domain, which is probably responsible for the additional activity of bradykinin potentiation. We propose the name CYLIP (cytotoxic linear peptide) to refer to the collective group of these peptides. While they have been documented as being lethal, the precise role of the scorpion cytolytic peptides in the envenoming function remains to be elucidated, just as is the case for enigmatic toxins such as nerve growth factors and hyaluronidases in snake venoms [81,82].

#### 2.2.4. Unravelling the Dynamic Molecular Evolution of Scorpion Toxins

**Figure 6:**
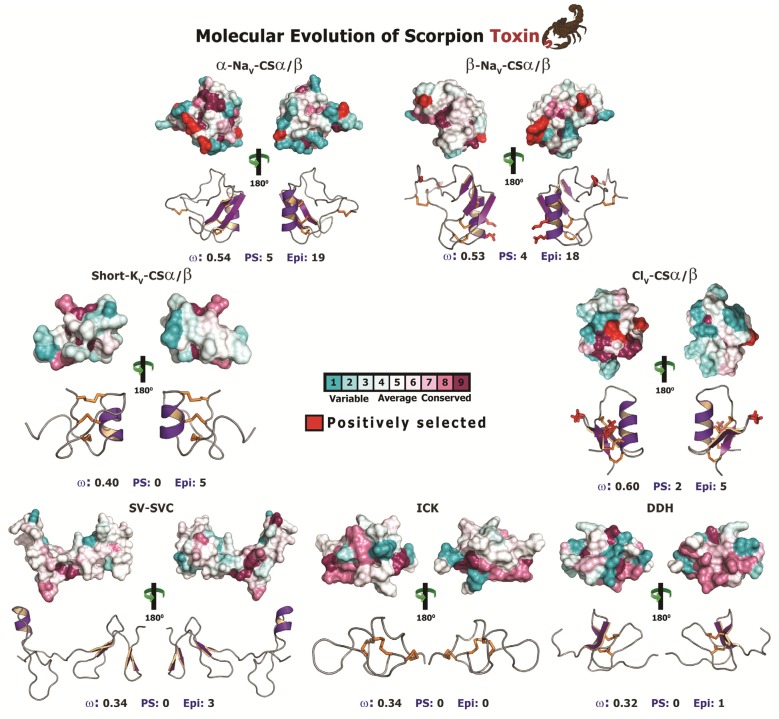
Molecular evolution of scorpion toxins. Three dimensional homology models of various scorpion CSα/β and non-CSα/β toxins, depicting the locations of positively selected sites are presented. Site-model 8 computed omega and the total number of positively selected sites (PS) detected by its Bayes Empirical Bayes (BEB) approach (*PP* ≥ 0.95) are indicated, along with the number of episodically diversifying sites (Epi) detected by MEME (at 0.05 significance). PDB codes used for modelling are: α-Na_V_-CSα/β: 1DJT; β-Na_V_-CSα/β: 2I61; Cl_V_-CSα/β: 1SIS; DDH: 2KYJ; ICK: 1IE6; short-K_V_-CSα/β: 1PVZ and SVC: 1U5M).

Site-specific models, implemented in codeml of the PAML package [83], failed to detect the influence of positive selection on the evolution of most scorpion toxin types (Table 3 and Table 4; Figure 6; Supplementary Tables 2.1–2.7). Only the plesiotypic Na_V_-CSα/β toxins were found to be positively selected, having an overall omega of more than 1 (ω = 1.63; 6 positively selected sites; Table 3). Although widely used for selection assessment, site-specific analyses are known to be influenced by sequence divergence in datasets [84,85,86] and often fail to detect episodic adaptations [87]. Not-surprisingly, site-specific assessments were only able to detect a handful of positively selected sites in very few CSα/β toxins: 6 in the plesiotypic-Na_V_-CSα/β; 2 in the lipolytic-Na_V_-CSα/β; 5 in the α-Na_V_-CSα/β; 4 in the β-Na_V_-CSα/β; and 2 in the Cl_V_-CSα/β) (Table 4; Figure 6; Supplementary Tables 3.1–3.8). In contrast, no positively selected sites were detected in any of the non-CSα/β toxins. Moreover, the computed ω values for the non-CSα/β toxins were extremely low and ranged from 0.14 to 0.34 (Table 4), indicating the significant role of negative selection in the evolution of non-CSα/β toxins.

**Table 3:** Molecular evolution of scorpion CSαβ toxins.

	FUBAR^a^	MEME^b^	BSR^c^	PAML^d^	
				M8	M2a
**Plesiotypic Na_V_-CSα/β**	ω > 1^e^ : 2 ω < 1^f^ : 11 ω > 1^e^ : 0 ω < 1^f^ : 20	5	4	6	5
				(3 + 3)	(3 + 2)
				1.63	1.76
**Lipolytic Na_V_-CSα/β**		2	1	2	5
				(2 + 0)	(2 + 3)
				0.76	1.28
**α-Na_V_-CSα/β**	ω > 1^e^ : 5 ω < 1^f^ : 33	19	14	5	5
				(0 + 5)	(1 + 4)
				0.54	0.8
**β-Na_V_-CSα/β**	ω > 1^e^ : 0 ω < 1^f^ : 37	18	16	4	8
				(2 + 2)	(6 + 2)
				0.53	1.03
**Long-K_V_-CSα/β**	ω > 1^e^ : 0 ω < 1^f^ : 57	10	2	0	0
				0.29	0.9
**Short-K_V_-CSα/β**	ω > 1^e^ : 1 ω < 1^f^ : 28	4	1	0	5
					(3 + 2)
				0.4	1.02
**Cl_V_-CSα/β**	ω > 1^e^ : 0 ω < 1^f^ : 10	5	2	2	0
				(0 + 2)	
				0.6	0.62

Legend: **a:** Fast Unconstrained Bayesian AppRoximation; **b:** Sites detected as experiencing episodic diversifying selection (0.05 significance) by the Mixed Effects Model Evolution (MEME); **c:** Number of branches detected by the branch-site REL (Random effects likelihood) test as episodically diversifying; **d:** Positively selected sites detected by the Bayes Empirical Bayes approach implemented in M8 and M2a. Sites detected at 0.99 and 0.95 significance are indicated in the parenthesis; **e:** number of sites under pervasive diversifying selection at the posterior probability ≥0.9 (FUBAR); **f:** Number of sites under pervasive purifying selection at the posterior probability ≥0.9 (FUBAR); **ω:** mean dN/dS.

**Table 4:** Molecular evolution of scorpion non-CSαβ toxins.

	FUBAR^a^	MEME^b^	BSR^c^	PAML^d^	
				M8	M2a
**SV-SVC**	ω > 1^e^ : 2 ω < 1^f^ : 32	3	0	0	0
				0.34	0.68
**ICK**	ω > 1^e^ : 0 ω < 1^f^ : 12	0	0	0	0
				0.34	0.49
**DDH**	ω > 1^e^ : 0 ω < 1^f^ : 6	1	0	0	0
				0.32	0.32
**AMP**	ω > 1^e^ : 1 ω < 1^f^ : 34	2	0	1	0
				(0 + 1)	
				0.33	0.34
**Linear**	ω > 1^e^ : 0 ω < 1^f^ : 45	3	0	0	0
				0.27	0.42
**Bradykinin**	ω > 1^e^ : 0 ω < 1^f^ : 36	2	0	0	0
				0.2	0.22
**Anionic**	ω > 1^e^ : 0 ω < 1^f^ : 33	0	0	0	0
				0.22	0.27
**Glycine-rich**	ω > 1^e^ : 0 ω < 1^f^ : 22	1	1	0	0
				0.14	0.35

Legend: **a:** Fast Unconstrained Bayesian AppRoximation; **b:** Sites detected as experiencing episodic diversifying selection (0.05 significance) by the Mixed Effects Model Evolution (MEME); **c:** Number of branches detected by the branch-site REL (Random effects likelihood) test as episodically diversifying; **d:** Positively selected sites detected by the Bayes Empirical Bayes approach implemented in M8 and M2a. Sites detected at 0.99 and 0.95 significance are indicated in the parenthesis; **e:** number of sites under pervasive diversifying selection at the posterior probability ≥0.9 (FUBAR); **f:** Number of sites under pervasive purifying selection at the posterior probability ≥0.9 (FUBAR); **ω:** mean dN/dS.

We further employed Fast, Unconstrained Bayesian AppRoximation (FUBAR [88]), which supersedes methods such as Single Likelihood Ancestor Counting (SLAC), Fixed Effects Likelihood (FEL) and Random Effects Likelihood (REL) [89], and detects sites evolving under the influence of pervasive diversifying and purifying selection pressures. FUBAR detected very few sites in both CSα/β and non-CSα/β scorpion toxin types (Table 3 and Table 4). Subsequently, additional support for the sites detected as positively selected by the nucleotide-specific analyses and the radicalness of non-synonymous replacements at these hypermutable sites was assessed by utilising an amino acid-level selection assessment method implemented in TreeSAAP [90], which measures selective influences on 31 structural and biochemical amino acid properties. Using this complementary nucleotide and amino acid-level approach, we were able to identify several sites in most CSα/β toxins as accumulating radical amino acid replacements (Table 5), which can alter the fitness of the organism by influencing toxin structure and function.

Evolutionary fingerprint analyses highlighted a small proportion of sites in plesiotypic-Na_V_-CSα/β, α-Na_V_-CSα/β and Cl_V_-CSα/β as evolving under the influence of positive selection, while a major proportion of sites in all other CSα/β and non-α/β toxin types were depicted as evolving under the strong influence of negative selection (Supplementary Figure 1 and Figure 2). We also employed the Branch-site REL test [91] to identify lineages that have experienced episodic bursts of adaptive selection. This test detected several branches in CSα/β scorpion toxin lineages as evolving under the influence of episodic diversifying selection pressures (plesiotypic-Na_V_-CSα/β: 4; lipolytic-Na_V_-CSα/β: 1; α-Na_V_-CSα/β: 14; β-Na_V_-CSα/β: 16; long-K_V_-CSα/β: 1; short-K_V_-CSα/β: 1; and Cl_V_-CSα/β: 2; Table 3; Supplementary Figures 3–9). In contrast, this test failed to identify episodically diversifying branches in all but glycine-rich (*n* = 1) non-CSα/β scorpion toxin lineages (Table 4), further highlighting that these toxins evolve under the strong influence of negative selection.

It should be noted that site-specific assessments assume that the strength of selection remains constant across all lineages over time, which is not always biologically justified, and fail to identify rapidly evolving sites when a large number of sites follow the regime of negative selection [87]. In contrast, genes could experience episodic bursts of adaptive selection pressures [87]. Scorpions are well known for their low dispersal rate and have evolved over 400 million years [14]. Combined with rapid time to sexual maturity, the toxin-encoding genes responsible for scorpion venom arsenal have greatly diversified over long periods of evolutionary time. Therefore to address these shortcomings, we employed the Mixed Effects Model Evolution (MEME), which is known to reliably and accurately capture the molecular footprints of both episodic and pervasive diversifying selection [87]. MEME identified a large number of episodically diversifying sites in most scorpion CSα/β toxins: 5 (6% sites) in plesiotypic-Na_V_-CSα/β; 2 (2% sites) in lipolytic-Na_V_-CSα/β; 19 (22% sites) in α-Na_V_-CSα/β; 18 (22% sites) in β-Na_V_-CSα/β; 8 (8% sites) in long-K_V_-CSα/β; 5 (8% sites) in short-K_V_-CSα/β; and 5 (8% sites) in Cl_V_-CSα/β (Table 3; Figure 6), and thus indicated that episodic adaptive selection pressures sculpt CSα/β toxin scaffolds in scorpions. In contrast, this test failed to detect any episodically diversifying sites in ICK and Anionic toxin types, while detecting very few episodically diversifying sites in other non-CSα/β toxins: 3 (3% sites) in SV-SVC; 1 (1% sites) in DDH; 2 (3% sites) in ‘AMP’ type CYLIP; 3 (4% sites) in ‘Linear’ type CYLIP; 2 (2% sites) in ‘BPP’ type CYLIP; and 1 (1% sites) in Glycine-rich peptides (Table 4; Figure 6). Thus, both site-specific assessments and MEME suggested that non-CSα/β toxins have evolved under the extreme influence of negative selection.

Thus, although the widely used conventional site-specific methods for identifying the nature of selection, failed to detect the influence of positive Darwinian selection on the evolution of scorpion venom, state-of-art molecular evolutionary assessments (MEME and BSR test) that are designed to overcome the shortcomings of site-specific methods detected several positively selected sites and branches in CSα/β toxins (particularly: α-, β-, plesiotypic Na_V_-, long-K_V_- and Cl_V_-CSα/β) as evolving under episodic bursts of selection and highlighted the dynamic molecular evolution of scorpion venom. It is noteworthy that not all CSα/β toxins appear to have undergone rapid evolution. Selection analyses (Table 3) in this study detected very few episodically diversifying sites in the lipolytic toxins (2% sites), which could be a result of their non-specific mechanism of action or the fact that they target non-plastic molecular targets in prey animals. Similarly, these highly sensitive methods failed to detect the influence of positive selection on the evolution of linear non-CSα/β toxins (0%–4% of sites; Table 4), which are secreted in large quantities by the non-buthid scorpion lineages [22,35,67,92]. Thus, genes encoding non-CSα/β toxins appeared to have followed the regime of negative selection. Unlike their homologues in spiders and cone snails, scorpion ICKs are known to target only RyR ion channels, with a single report of K^+^ ion channel targeting activity [49]. Since scorpions have recruited a diversity of other scaffolds to target K^+^ ion channels [13,93], it has been theorized that scorpion ICK peptides targeting K^+^ ion channels could exhibit weak potency [49]. Therefore, ICKs are considered to play only an ancillary role in scorpion envenoming [49]. It should also be noted that the role of these toxins as mammalian RyR activators and whether they serve an envenoming role remains unexplored to date. Surprisingly, selection assessments conducted in this study revealed that ICK toxins have evolved under extreme constraints of negative selection. The lack of variation in their coding sequences, despite the fact that they have evolved over many millions of years, suggests that they probably play an important role in scorpion venom arsenal. The lack of variation may also suggest that they attack extremely well conserved molecular targets, and as a result do not experience a coevolutionary arms race. The fact that scorpions have recruited several toxin scaffolds to target K_V_ ion channels could have also lead to a decreased selection pressure for accumulating variations in K_V_ targeting toxins.

#### 2.2.5. Focal Mutagenesis Shapes the Molecular Evolution of Scorpion CSα/β Toxin Families

**Figure 7:**
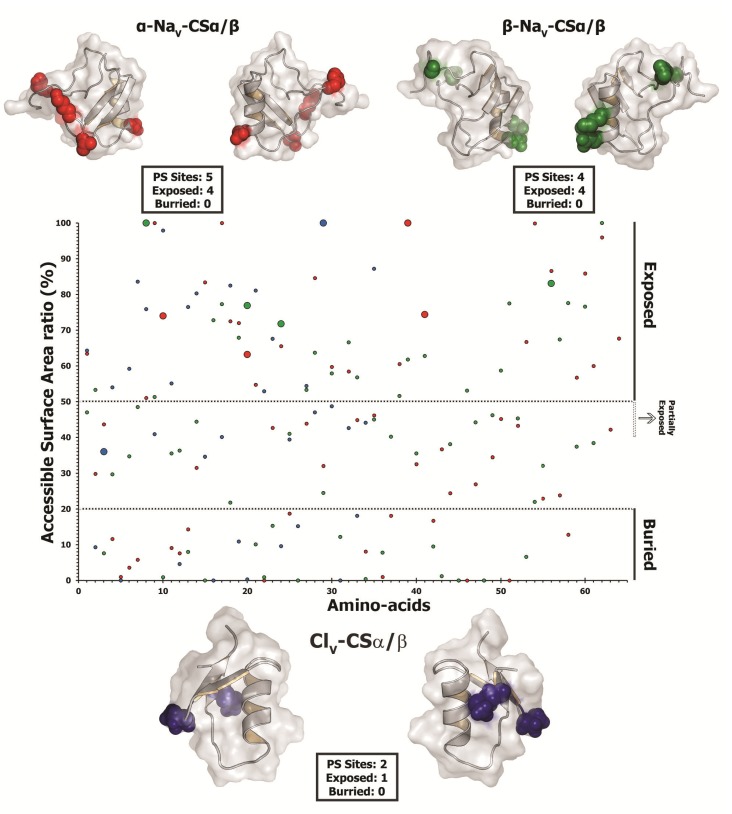
Surface accessibility of hypermutable sites. A plot of amino acid positions (x-axis) against accessible surface area (ASA) ratio (y-axis) indicating the locations of amino acids (exposed or buried) in the crystal structure of various scorpion toxins is presented. Positively selected residues are presented as large dots, while the remaining sites are presented as small dots in the plot. Residues with an ASA ratio greater than 50% are considered to be exposed, while those with an ASA ratio less than 20% are considered to be buried to the surrounding medium (ASA of 21%–39%: cannot be assigned to buried/exposed class; ASA of 40%-50% are likely to have exposed side chains). Three dimensional homology models of various scorpion toxin types, depicting the locations of positively selected (PS) sites along with model 8 omega values and the number of exposed and buried positively selected sites are also presented. PDB codes used for modelling are: α-Na_V_-CSα/β: 1DJT; β-Na_V_-CSα/β: 2I61; Cl_V_-and CSα/β: 1SIS.

The state-of-the-art molecular selection assessments presented in this study clearly highlight the significant role of point-mutations, which episodically accumulate under the influence of positive Darwinian selection, in sculpting the diversity of CSα/β scorpion toxins. A diversity of venom-components in a wide range of venomous lineages—such as, spiders, scorpions, snakes, lizards, coleoids (cuttlefish, octopus and squid), vampire bats, *etc.*, have been shown to adopt RAVER (Rapid Accumulation of Variations in Exposed Residues), a phenomenon where focal mutagenesis favours the accumulation of point mutations via positive selection in certain regions of predatory toxins, such as the molecular surface and the loops of the toxin [1,2,12,81,94,95,96,97,98,99]. As a result of this intriguing evolutionary phenomenon a diversity of residues are generated on the molecular surface of the toxin, which may interact with novel cell receptors when injected into prey animals. Despite favouring accumulation of variations, focal mutagenesis would ensure the conservation of structurally and functionally important core residues in enzymatic toxins. After all, the synthesis and secretion of venom-components is an energetically expensive process [100]. Hence, focal mutagenesis ensures the accumulation of essential variation in venom, while alleviating the risk of secreting faulty enzymes. In certain organisms like vampire bats, focal mutagenesis in venom-components can be advantageous as it is likely to delay/prevent the development of immunological resistance in the prey by introducing extremely variable toxin surface chemistry in the vampire bat population [12]. Evidently, it has been demonstrated that prolonged targeting and feeding of prey-animals by vampire bats leads to the development of immunological resistance in the prey against venom-components like draculin [101]. Mapping of mutations on the three-dimensional structures and computation of accessible surface area for scorpion CSα/β toxins revealed that a large proportion of hypermutable sites have accumulated on the molecular surfaces: four/five sites in α-Na_V_-CSα/β; all four sites in β-Na_V_-CSα/β; and one/two sites in Cl_V_-CSα/β. One site each in α-Na_V_-CSα/β and Cl_V_-CSα/β couldn’t be assigned to exposed/buried class (Table 5; Figure 7). Moreover, amino acid-level selection assessments indicated that these non-synonymous replacements introduced radical changes in the amino acid properties on the molecular surface of toxins (Table 5). Such toxins with extremely variable surface chemistry could interact with novel molecular targets of the prey animals. Thus, focal mutagenesis has played a significant role in the evolution of predatory peptide toxins in scorpion lineages. We theorize that RAVER plays a significant role in the divarication and neofunctionalisation of predatory animal toxins.

**Table 5:** Nucleotide and complementary amino acid-level selection assessment of CSα/β toxins.

Site		CodeML		TreeSAAP		ASA(%)
Codon	AA	M2a ^a^	M8 ^b^	Property ^c^	Magnitude ^d^	
Long 3CC						
**33**	**D**	**5.858 ± 1.374**	**4.775 ± 1.168**	***P*_b_, α*_C_***	**7, 8**	
		(0.997)**	(0.998)**			
**42**	**N**	**5.752 ± 1.531**	**4.726 ± 1.233**	***V°***	**6**	
		(0.975)*	(0.986)*			
**47**	**K**	**5.827 ± 1.427**	**4.761 ± 1.189**	***V°***	**6**	
		(0.990)**	(0.995)**			
**51**	**D**	**5.703 ± 1.587**	**4.707 ± 1.253**	***V°***	**6**	
		(0.966)*	(0.982)*			
**65**	**Y**	**5.870 ± 1.356**	**4.78 ± 1.162**	***P*_b_, *V°*, α*_C_*, *E_SM_***	**7, 6, 6, 6**	
		(0.999)**	(0.999)**			
**75**	**D**	**5.580 ± 1.740**	**4.643 ± 1.331**	***P*_b_**	**7**	
		**−0.941**	(0.966)*			
Lipolysis Activating						
61	H	4.678 ± 0.900	3.042 ± 0.857	-	-	-
		(1.000)**	(0.997)**			
**81**	**S**	**4.672 ± 0.914**	**3.026 ± 0.867**	**α*_C_***	**6**	-
		(0.998)**	(0.991)**			
Alpha NaScTx						
**29**	**E**	**2.427 ± 0.324**	**1.471 ± 0.136**	***pHi, E_l_ , Mv,* α*_C_*, *E_SM_***	**8, 8, 7, 7, 6**	**74**
		(0.952)*	(0.950)*			**Exposed**
**40**	**E**	**2.463 ± 0.233**	**1.473 ± 0.133**	***pHi, E_l_, Mv,* α*_C_*, *E_SM_***	**8, 8, 7, 7, 6**	**63.2**
		(0.976)*	(0.955)*			**Exposed**
**59**	**G**	**2.464 ± 0.232**	**1.478 ± 0.121**	***Mv*, *E_SM_***	**7, 6**	**100**
		(0.977)*	(0.962)*			**Exposed**
**61**	**K**	**2.491 ± 0.122**	**1.492 ± 0.083**	***E_l_ , Mv*, *E_SM_***	**8, 7, 6**	**74.4**
		(0.994)**	(0.985)*			**Exposed**
**85**	**R**	**2.439 ± 0.297**	**1.472 ± 0.135**	***Mv***	**7, 6**	
		(0.960)*	(0.952)*			
Beta NaScTx						
**27**	**S**	**2.500 ± 0.001**	**1.498 ± 0.035**	***P*_a_, *K°, pK^!^***	**6, 6, 8**	**100**
		(1.0)**	(0.996)**			**Exposed**
**39**	**E**	**2.500 ± 0.005**	**1.487 ± 0.084**	***P*_a_, *K°***	**6, 8**	**76.9**
		(1.0)**	(0.978)*			**Exposed**
**43**	**K**	**2.500 ± 0.001**	**1.497 ± 0.043**	***P*_a_, *K°***	**6, 8**	**71.8**
		(1.0)**	(0.994)**			**Exposed**
**72**	**L**	**2.500 ± 0.003**	**1.493 ± 0.062**	***K°, pK^!^, F***	**8, 8, 8**	**83.1**
		(1.0)**	(0.988)*			**Exposed**
Chloride ion-channel Toxin						
**27**	**P**	**1.833 ± 0.844**	**1.521 ± 0.223**	**α*_C_*, *R*_a_**	**6, 7**	**36**
		**-0.814**	(0.984)*			**NA**
**53**	**Y**	**1.792 ± 0.963**	**1.508 ± 0.244**	***R*_a_**	**7**	**100**
		**−0.737**	(0.966)*			**Exposed**

Amino-acid property symbols used: α-helical tendencies (Pa), β-structure tendencies (Pb), Compressibility (K°), Equilibrium constant (ionization of COOH) (pK1), Isoelectric point (pHi), Long-range non-bound energy (El), Mean R.M.S. fluctuation displacement (F), Molecular volume (Mv), Partial specific volume (V°), Power to be at the C-terminus of α-helix (αC), Short and medium-range energy (Esm) and Solvent accessible reduction ratio (Ra). **Legend: a**: M2a Bayes Empirical Bayes (BEB) posterior probability (* ≥ 0.95; ** ≥ 0.99) and post-mean omega indicated in brackets; **b**: M8 Bayes Empirical Bayes (BEB) posterior probability (* ≥ 0.95; ** ≥ 0.99) and post-mean omega indicated in brackets; **c**: amino acid property under selection; **d**: magnitude of selection on the amino acid property; **ASA**: Accessible surface area (50% ≥ Side chains completely exposed; 20% ≤ Side chains buried); **Part. exposed**: Partially exposed side-chains (ASA: 40%–50%). Sites detected as positively selected by both nucleotide and amino acid-level analyses are indicated in bold.

## 3. Experimental Section

### 3.1. Specimens

BOTHRIURIDAE

*Cercophonius squama* (Wauchope, New South Wales) 

BUTHIDAE

*Australobuthus xerlomnion* (Lake Gardner, South Australia) 

*Isometroides vescus* (Port Pirie, South Australia) 

*Lychas buchari* (Port Pirie, South Australia) 

URODACIDAE

*Urodacus manicatus* (Traralgon, Victoria).

### 3.2. Transcriptome Library Construction

In order to minimise individual or time-course variation, 4–6 days post-milking, six telsons for each species were dissected out (two per day) and pooled and immediately frozen in liquid nitrogen for future use. Total RNA extracted using the standard TRIzol Plus method (Invitrogen). Extracts were enriched for mRNA using standard RNeasy mRNA mini kit (Qiagen) protocol. mRNA was reverse transcribed, fragmented and ligated to a unique 10-base multiplex identifier (MID) tag prepared using standard protocols and applied to one PicoTitrePlate (PTP) for simultaneous amplification and sequencing on a Roche 454 GS FLX+ Titanium platform (Australian Genome Research Facility). Automated grouping and analysis of sample-specific MID reads informatically separated sequences from the other transcriptomes on the plates, which were then post-processed to remove low quality sequences before de novo assembly into contiguous sequences (contigs) using v3.4.0.1 of the MIRA software program. Assembly details are provided in Supplementary Table 1. Public access of the data can be found at the National Center for Biotechnology Information (NCBI) under bioprojects: *Australobuthus xerlomnion PRJNA201348; Cercophonius squama* PRJNA201349; *Isometroides vescus PRJNA201350; Lychas buchari PRJNA201351*; *Urodacus manicatus PRJNA201352*. Sequenced analysed in this study have the accession numbers of: *Australobuthus xerlomnion GALG01000001-GALG01000002*; *Cercophonius squama* GALH01000001-GALH01000013; *Isometroides vescus GALK01000001-GALK01000018*; *Lychas buchari GALL01000001-GALL01000032*; *Urodacus manicatus GALI01000001-GALI01000019*. Sequences are also directly available in Supplementary File 1.

Assembled contigs were processed using CLC Main Work Bench (CLC-Bio) and Blast2GO bioinformatic suite [102,103] to provide Gene Ontology, BLAST and domain/Interpro annotation. The above analyses assisted in the rationalisation of the large numbers of assembled contigs into phylogenetic ‘groups’ for detailed phylogenetic analyses outlined below that focused upon the peptide toxin types.

### 3.3. Sequence Retrieval and Alignment

Amino acid sequences were aligned in CLC Main Work Bench (CLC-Bio) using default parameters and were then edited manually to align any misaligned region [104]. Amino acid alignments generated this way were used for guiding the nucleotide alignments, which were trimmed before conducting selection analyses to remove regions with gaps in more than 50% of sequences. Nucleotides and the translated nucleotide alignments used for selection assessments are presented in Supplementary file 1.

### 3.4. Phylogenetics

The molecular evolutionary histories of various scorpion toxins were reconstructed using phylogenetic analyses. Trees were generated using the Bayesian inference implemented in Mr. Bayes 3.2.1 [105], which is well known for its ability to deal with divergent datasets. The command block lset rates = invgamma with prset aamodelpr = mixed was used, which enables the program to optimize between nine different amino acid substitution matrices implemented in Mr. Bayes. The analysis was performed by running a minimum of 1 × 10^7^ generations in four chains, and saving every 100th tree. The log-likelihood score of each saved tree was plotted against the number of generations to establish the point at which the log likelihood scores reached their asymptote (stationarity), and the posterior probabilities for clades established by constructing a majority-rule consensus tree for all trees generated after completion of the burn-in phase. Optimal maximum likelihood phylogenetic trees, used in selection assessments, were obtained using PhyML 3.0 [106] and node support was evaluated with 1,000 bootstrapping replicates (Supplementary Figures 3–9). Amino acid alignments used for reconstructing the Bayesian trees are presented in the supplementary material.

### 3.5. Test for Recombination

To overcome the effects of recombination on the molecular evolution interpretations we employed Single Breakpoint algorithm implemented in the HyPhy package and assessed the effect of recombination on all the toxin types examined in this study [107]. When potential breakpoints were detected using the small sample Akaike information Criterion (AICc), the sequences were portioned before conducting selection analyses to allow recombining units to have distinct phylogenetic histories [108]. 

### 3.6. Selection Analyses

The influence of natural selection on various scorpion toxin types was evaluated using the maximum-likelihood models [109,110] implemented in CODEML of the PAML [83]. We employed site-specific models that estimate positive selection statistically as a non-synonymous-to-synonymous nucleotide-substitution rate ratio (ω) significantly greater than 1. We compared likelihood values for three pairs of models with different assumed ω distributions as no a priori expectation exists for the same: M0 (constant ω rates across all sites) *versus* M3 (allows the ω to vary across sites within ‘n’ discrete categories, *n* ≥ 3); M1a (a model of neutral evolution) where all sites are assumed to be either under negative (ω < 1) or neutral selection (ω = 1) *versus* M2a (a model of positive selection) which in addition to the site classes mentioned for M1a, assumes a third category of sites; sites with ω > 1 (positive selection) and M7 (Beta) *versus* M8 (Beta and ω), and models that mirror the evolutionary constraints of M1 and M2 but assume that ω values are drawn from a beta distribution [111]. Only if the alternative models (M3, M2a and M8: allow sites with ω > 1) show a better fit in Likelihood Ratio Test (LRT) relative to their null models (M0, M1a and M7: do not allow sites ω > 1), are their results considered significant. LRT is estimated as twice the difference in maximum likelihood values between nested models and compared with the χ^2^ distribution with the appropriate degree of freedom—the difference in the number of parameters between the two models. The Bayes empirical Bayes (BEB) approach [112] was used to identify codon sites under positive selection by calculating the posterior probabilities that a particular amino acid belongs to a given selection class (neutral, conserved or highly variable). Sites with greater posterior probability (*PP* ≥ 95%) of belonging to the ‘ω > 1 class’ were inferred to be positively selected.

Fast, Unconstrained Bayesian AppRoximation (FUBAR) approach implemented in HyPhy [88,113] was employed to provide additional support to the aforementioned analyses and to detect sites evolving under the influence of pervasive diversifying and purifying selection. Mixed Effects Model Evolution (MEME) [87] was also used to detect episodic diversifying selection. We utilised the branch-site REL test [91] to detect lineages in scorpion toxin phylogenies that evolve under the influence of episodic adaptation. To derive further support for the sites detected as positively selected by the nucleotide-level selection analyses, we employed a complementary protein-level approach implemented in TreeSAAP [90]. To clearly depict the proportion of sites under different regimes of selection, an evolutionary fingerprint analysis was carried out using the ESD algorithm implemented in datamonkey [114].

### 3.7. Structural Analyses

To depict the natural selection pressures influencing the evolution of various three-finger toxins, we mapped the sites under positive selection on the homology models created using Phyre 2 webserver [115]. Pymol 1.3 [116] was used to visualize and generate the images of homology models. Consurf webserver [117] was used for mapping the evolutionary selection pressures on the three-dimensional homology models. GETAREA [118] was used to calculate the Accessible Surface Area (ASA) or the solvent exposure of amino-acid side chains. It uses the atom co-ordinates of the PDB file and indicates if a residue is buried or exposed to the surrounding medium by comparing the ratio between side chain Accessible Surface Area (ASA) and the “random coil” values per residue. An amino-acid is considered to be buried if it has an ASA less than 20% and exposed if ASA is more than or equal to 50%. When ASA ratio lies between 40% and 50%, it is highly likely that the residues have their side chains exposed to the surrounding medium. PDB codes used in modelling were: α-Na_V_-CSα/β: 1DJT; β-Na_V_-CSα/β: 2I61; Cl_V_-CSα/β: 1SIS; DDH: 2KYJ; ICK: 1IE6; short-K_V_-CSα/β: 1PVZ and SVC: 1U5M).

## 4. Conclusion

We not only unravel the evolutionary origin of neglected scorpion toxin scaffolds (ICK, DDH, linear peptides) for the first time, but we have also highlighted the putative common origin of different cytotoxic peptides (AMP, linear and BPP). For the first time, we have discovered the plesiotypic form (SV-SVCs) of ICK and DDH toxins. In addition to being a fascinating system for molecular evolutionary studies, the documentation of much greater range of complexity/diversity in coding sequences of these toxins, which was previously undermined as a result of extreme divergence, underscores the tremendous opportunity for biodiscovery within scorpion venoms. A large number of toxin clades contain abundant sequences that are either under-investigated or remain entirely neglected by toxinological research. For instance, despite being the major venom-components in some species, peptide types like SV-SVCs remain functionally uncharacterised. Similarly, other peptide toxin types, such as anionic and glycine-rich toxins are yet to be functionally characterised. Even in the intensely investigated Na_V_-CSα/β clade, our results clearly demonstrate a rich biodiversity far beyond the traditional site-3 and site-4 toxins. In contrast to the molecular evolutionary patterns of cysteine-rich CSα/β toxins, some of which were documented to have adopted RAVER, all the non-CSα/β peptides were shown to be extremely negatively-selected. We hope that our work not only stimulates research interest into the complex molecular evolutionary history of scorpion venom peptides from a theoretical perspective, but also stimulates investigation into the structure-function relationships and functional characterisations of novel peptide clades and their potential utilisation in drug design and development.

## Supplementary Files

Supplementary File 1 Supplementary (ZIP, 4932 KB)
