# Supplementary material for: Evolution Stings: The Origin and Diversification of Scorpion Toxin Peptide Scaffolds
Source: Toxins (Basel). 2013 Dec 13;5(12):2456–87. doi: 10.3390/toxins5122456 (PMC3873696; doi:10.3390/toxins5122456)
Supplement: Supplementary File 1 — Supplementary (ZIP, 4932 KB) [file toxins-05-02456-s001.zip › Supplementary Figure 3 - Plesiotypic and Lipolytic Ntxs.pdf]

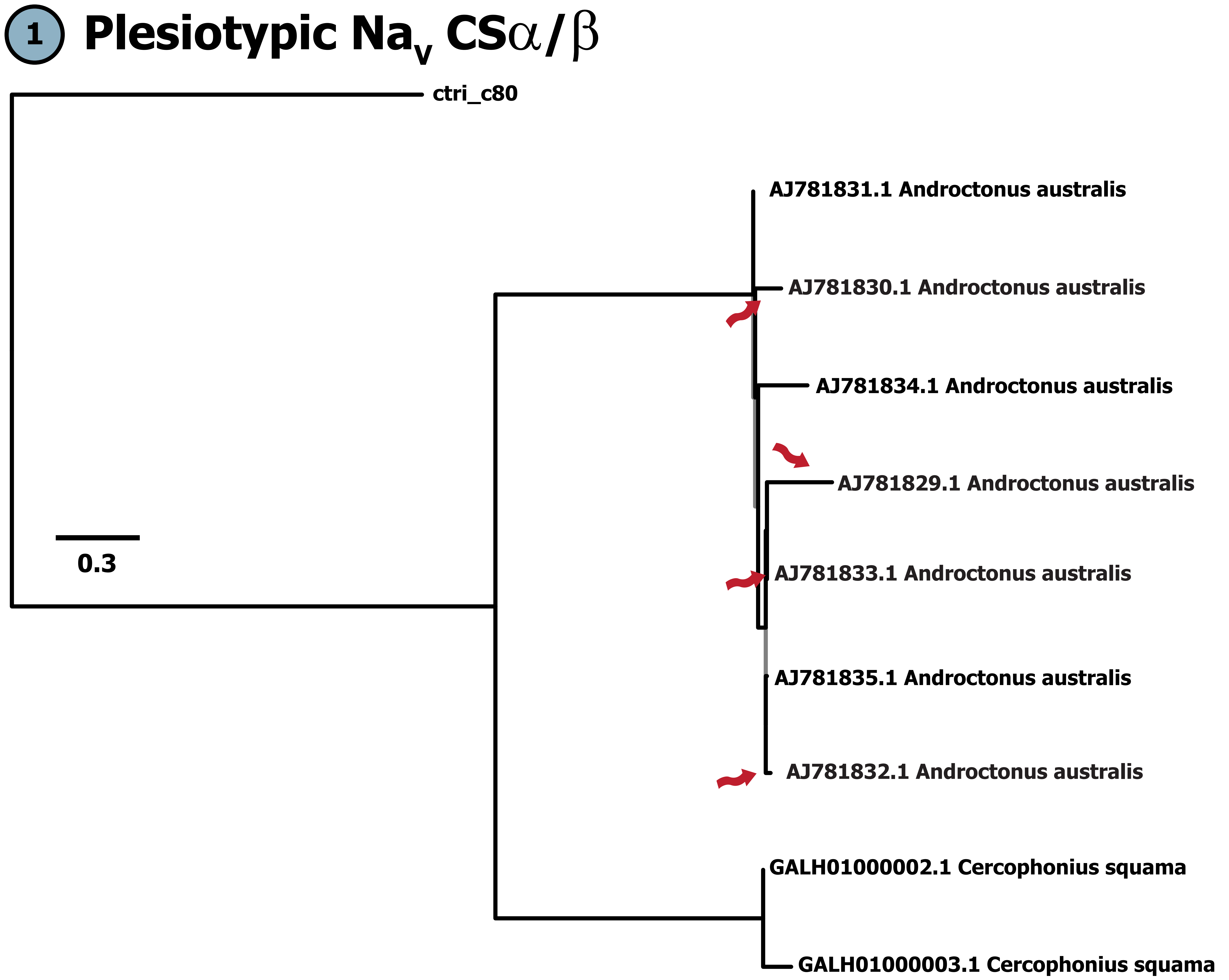
1
Plesiotypic Na_v CSα/β
ctri_c80
AJ781831.1 Androctonus australis
AJ781830.1 Androctonus australis
AJ781834.1 Androctonus australis
AJ781829.1 Androctonus australis
AJ781833.1 Androctonus australis
AJ781835.1 Androctonus australis
AJ781832.1 Androctonus australis
GALH01000002.1 Cercophonius squama
GALH01000003.1 Cercophonius squama
0.3

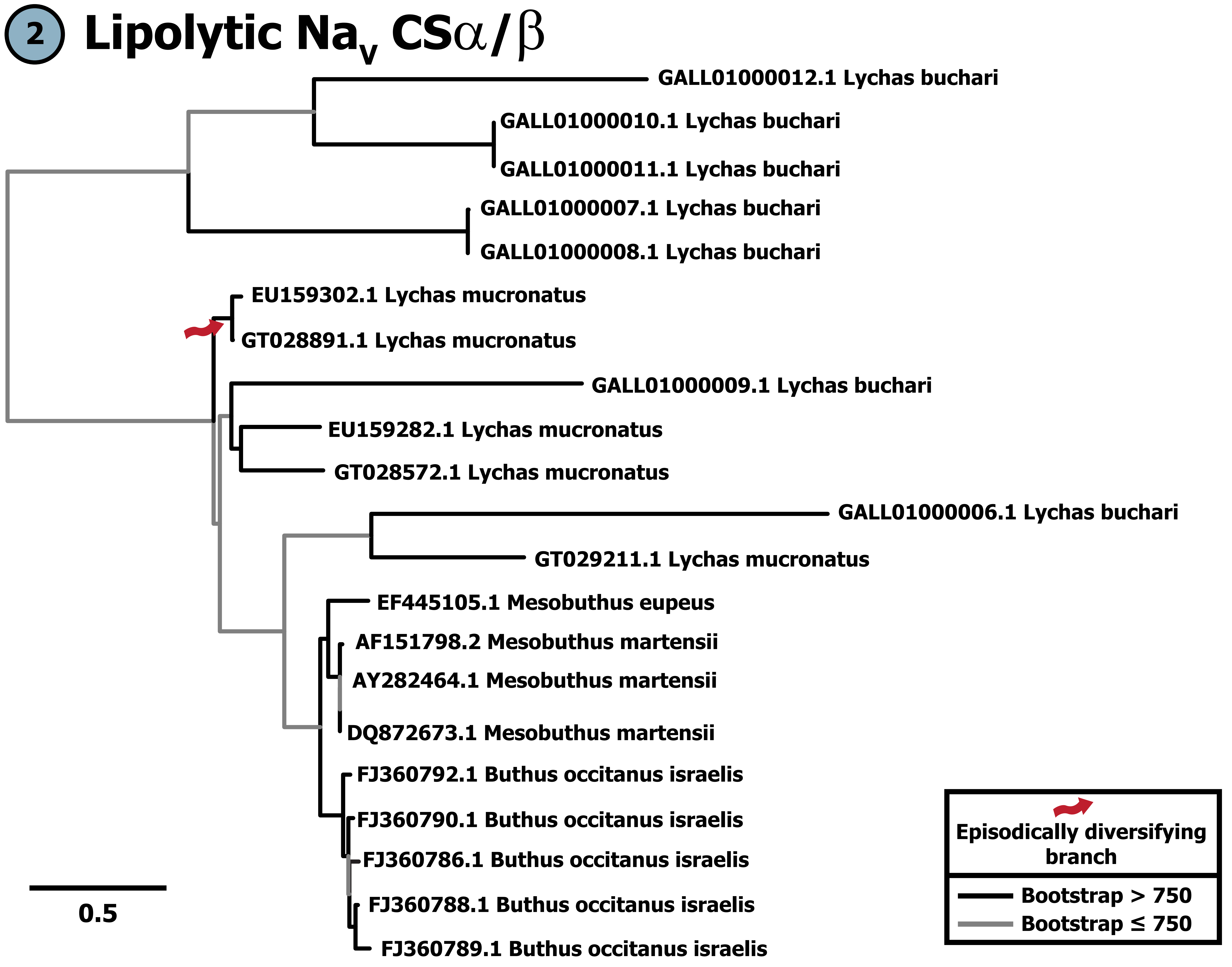
2
Lipolytic Na_v CSα/β
GALL01000012.1 Lychas buchari
GALL01000010.1 Lychas buchari
GALL01000011.1 Lychas buchari
GALL01000007.1 Lychas buchari
GALL01000008.1 Lychas buchari
EU159302.1 Lychas mucronatus
GT028891.1 Lychas mucronatus
GALL01000009.1 Lychas buchari
EU159282.1 Lychas mucronatus
GT028572.1 Lychas mucronatus
GALL01000006.1 Lychas buchari
GT029211.1 Lychas mucronatus
EF445105.1 Mesobuthus eupeus
AF151798.2 Mesobuthus martensii
AY282464.1 Mesobuthus martensii
DQ872673.1 Mesobuthus martensii
FJ360792.1 Buthus occitanus israelis
FJ360790.1 Buthus occitanus israelis
FJ360786.1 Buthus occitanus israelis
FJ360788.1 Buthus occitanus israelis
FJ360789.1 Buthus occitanus israelis
0.5
Episodically diversifying branch
Bootstrap > 750
Bootstrap ≤ 750

Note: As a standard practice, all HyPhy analyses are conducted using a dataset of unique sequences only (duplicates were removed). In addition, extremely divergent sequences present in the short KTx dataset were also removed prior to analyses. Full set of nucleotide sequences are available in Supplementary File 1, while all used sequences are shown in branch-site REL supplementary figures.
