# Supplementary material for: Evolution Stings: The Origin and Diversification of Scorpion Toxin Peptide Scaffolds
Source: Toxins (Basel). 2013 Dec 13;5(12):2456–87. doi: 10.3390/toxins5122456 (PMC3873696; doi:10.3390/toxins5122456)
Supplement: Supplementary File 1 — Supplementary (ZIP, 4932 KB) [file toxins-05-02456-s001.zip › Supplementary Figure 6 - Chloride.pdf]

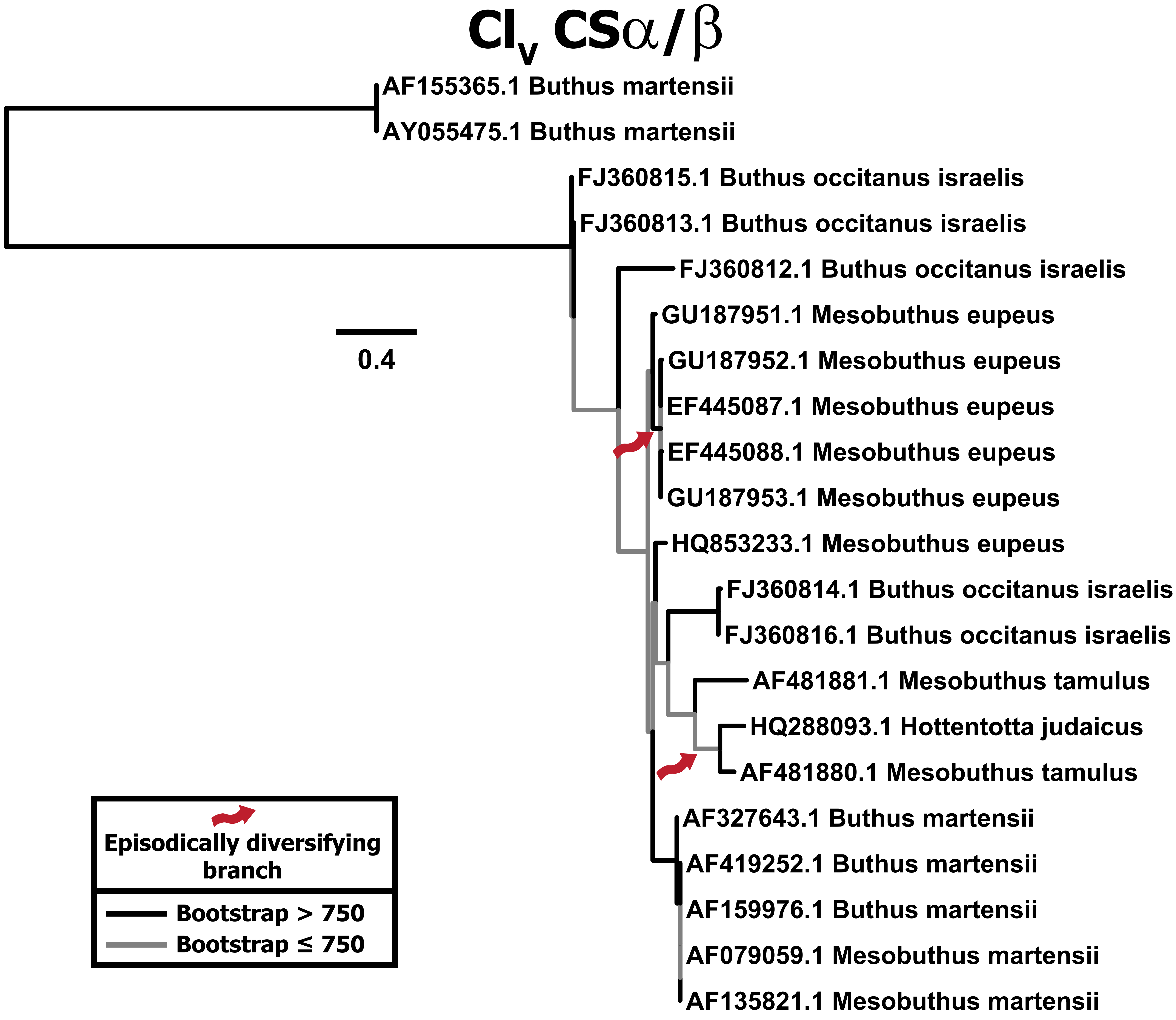

Cl_V CSα/β
AF155365.1 Buthus martensii
AY055475.1 Buthus martensii
FJ360815.1 Buthus occitanus israelis
FJ360813.1 Buthus occitanus israelis
FJ360812.1 Buthus occitanus israelis
GU187951.1 Mesobuthus eupeus
GU187952.1 Mesobuthus eupeus
EF445087.1 Mesobuthus eupeus
EF445088.1 Mesobuthus eupeus
GU187953.1 Mesobuthus eupeus
HQ853233.1 Mesobuthus eupeus
FJ360814.1 Buthus occitanus israelis
FJ360816.1 Buthus occitanus israelis
AF481881.1 Mesobuthus tamulus
HQ288093.1 Hottentotta judaicus
AF481880.1 Mesobuthus tamulus
AF327643.1 Buthus martensii
AF419252.1 Buthus martensii
AF159976.1 Buthus martensii
AF079059.1 Mesobuthus martensii
AF135821.1 Mesobuthus martensii
0.4
Episodically diversifying branch
Bootstrap > 750
Bootstrap ≤ 750
