# Supplementary material for: Evolution Stings: The Origin and Diversification of Scorpion Toxin Peptide Scaffolds
Source: Toxins (Basel). 2013 Dec 13;5(12):2456–87. doi: 10.3390/toxins5122456 (PMC3873696; doi:10.3390/toxins5122456)
Supplement: Supplementary File 1 — Supplementary (ZIP, 4932 KB) [file toxins-05-02456-s001.zip › Supplementary Table 1.docx]

**Supplementary Table 1:** Assembly details

|  | ***Australobuthus xerlomnion*** | ***Cercophonius***  ***squama*** | ***Isometroides vescus*** | ***Lychas***  ***buchari*** | ***Urodacus manicatus*** |
| --- | --- | --- | --- | --- | --- |
| **Reads** | 53 510 | 59 174 | 65 490 | 75 950 | 76 540 |
| **Total number of bases** | 20 833 026 | 2 151 356 | 22 911 959 | 27 265 869 | 26 715 502 |
| **Average read length** | 389 | 363 | 349 | 358 | 349 |
| **Contigs** | 3 531 | 2 733 | 2 856 | 5 534 | 6 884 |
| **Assembled bases** | 1 962 322 | 1 531 957 | 1 572 328 | 3 402 763 | 4 043703 |
| **Average contig length** | 556 | 561 | 551 | 615 | 587 |
