# Supplementary material for: Evolution Stings: The Origin and Diversification of Scorpion Toxin Peptide Scaffolds
Source: Toxins (Basel). 2013 Dec 13;5(12):2456–87. doi: 10.3390/toxins5122456 (PMC3873696; doi:10.3390/toxins5122456)
Supplement: Supplementary File 1 — Supplementary (ZIP, 4932 KB) [file toxins-05-02456-s001.zip › Supplementary Table 2.1-2.7 - Selection analyses details for CSab toxins.docx]

**Table 2.1** Maximum-likelihood parameter estimates for the plesiotypic-Na_V_-CSα/β

| Model | Likelihood () | ω_0_^a^ | Parameters | Sign.^b^ | No. of Sites with ω > 1^c^ |
| --- | --- | --- | --- | --- | --- |
|  |  |  |  |  | **B.E.B** |
|  |  |  |  |  |  |
| M0 (One ratio) | -1422.144334 | 0.70 | = ω_0_ |  | - |
|  |  |  |  |  |  |
| M1 (Neutral) | -1389.698490 | 0.80 | P_0_: 0.199  ω_0_: 0.03  P_1_: 0.800  ω1:1.0 | P << 0.001 | - |
| M2 (Selection)* | -1377.525107 | 1.76 | P_0_: 0.173  ω_0_: 0.02  P_1_: 0.623  ω_1_:1.0  P_2_: 0.202  ω_2_: 5.58 |  | 3 (PP ≥ 0.99)  2 (P ≥ 0.95) |
| M3 (Discrete)* | -1375.904522 | 1.38 | P_0_: 0.151  ω_0_: 0.004  P_1_: 0.543  ω_1_: 0.504  P_2_: 0.304  ω_2_: 3.64 | P << 0.001 | - |
| M7 (beta) | -1388.352434 | 0.68 | p: 0.32137  q: 0.15002 | P << 0.001 | - |
| M8 (beta and ω)* | -1377.101414 | 1.63 | p_0_: 0.793  p: 0.186  q: 0.072  p1: 0.206  ω: 5.16 |  | 3 (PP ≥ 0.99)  3 (P > 0.95) |
|  |  |  |  |  |  |

**Legend:**

**a:** dn/ds (weighted average)

**b:** Significance of the model in comparison with the null model

**c:** Number of sites with ω > 1 under the Bayes empirical Bayes approach with a posterior probability (PP) more than or equal to 0.99 and 0.95

***** Models which allow ω > 1

**Table 2.2** Maximum-likelihood parameter estimates for lipolytic Na_V_-CSα/β

| Model | Likelihood () | ω_0_^a^ | Parameters | Sign.^b^ | No. of Sites with ω > 1^c^ |
| --- | --- | --- | --- | --- | --- |
|  |  |  |  |  | **B.E.B** |
|  |  |  |  |  |  |
| M0 (One ratio) | -3607.638244 | 0.36 | = ω_0_ |  | - |
|  |  |  |  |  |  |
| M1 (Neutral) | -3469.452509 | 0.69 | P_0_: 0.329  ω_0_: 0.07  P_1_: 0.670  ω1: 1.0 | P << 0.001 | - |
| M2 (Selection)* | -3454.484594 | 1.28 | P_0_: 0.239  ω_0_: 0.041  P_1_: 0.613  ω_1_:1.0  P_2_: 0.146  ω_2_: 4.52 |  | 2 (PP ≥ 0.99)  3 (P ≥ 0.95) |
| M3 (Discrete)* | -3437.619302 | 0.65 | P_0_: 0.216  ω_0_: 0.01  P_1_: 0.457  ω_1_: 0.34  P_2_: 0.326  ω_2_: 1.51 | P << 0.001 | - |
| M7 (beta) | -3439.037919 | 0.48 | p: 0.39284  q: 0.41732 | P << 0.001 | - |
| M8 (beta and ω)* | -3431.204900 | 0.76 | p_0_: 0.877  p: 0.397  q: 0.487  p1: 0.122  ω: 3.02 |  | 2 (PP ≥ 0.99)  0 (P > 0.95) |
|  |  |  |  |  |  |

**Legend:**

**a:** dn/ds (weighted average)

**b:** Significance of the model in comparison with the null model

**c:** Number of sites with ω > 1 under the Bayes empirical Bayes approach with a posterior probability (PP) more than or equal to 0.99 and 0.95

***** Models which allow ω > 1

**Table 2.3** Maximum-likelihood parameter estimates for α-Na_V_-CSα/β

| Model | Likelihood () | ω_0_^a^ | Parameters | Sign.^b^ | No. of Sites with ω > 1^c^ |
| --- | --- | --- | --- | --- | --- |
|  |  |  |  |  | **B.E.B** |
|  |  |  |  |  |  |
| M0 (One ratio) | -5750.454453 | 0.39 | = ω_0_ |  | - |
|  |  |  |  |  |  |
| M1 (Neutral) | -5568.078976 | 0.62 | P_0_: 0.431  ω_0_: 0.14  P_1_: 0.568  ω1: 1.0 | P << 0.001 | - |
| M2 (Selection)* | -5556.365346 | 0.80 | P_0_: 0.407  ω_0_: 0.14  P_1_: 0.465  ω_1_:1.0  P_2_: 0.126  ω_2_: 2.24 |  | 1 (PP ≥ 0.99)  4 (P ≥ 0.95) |
| M3 (Discrete)* | -5524.643103 | 0.51 | P_0_: 0.252  ω_0_: 0.04  P_1_: 0.502  ω_1_: 0.41  P_2_: 0.244  ω_2_: 1.21 | P << 0.001 | - |
| M7 (beta) | -5519.922419 | 0.45 | p: 0.50184  q: 0.60632 | P << 0.001 | - |
| M8 (beta and ω)* | -5512.987233 | 0.54 | p_0_: 0.870  p: 0.593  q: 0.942  p1: 0.129  ω: 1.60 |  | 0 (PP ≥ 0.99)  5 (P > 0.95) |
|  |  |  |  |  |  |

**Legend:**

**a:** dn/ds (weighted average)

**b:** Significance of the model in comparison with the null model

**c:** Number of sites with ω > 1 under the Bayes empirical Bayes approach with a posterior probability (PP) more than or equal to 0.99 and 0.95

***** Models which allow ω > 1

**Table 2.4** Maximum-likelihood parameter estimates for β-Na_V_-CSα/β

| Model | Likelihood () | ω_0_^a^ | Parameters | Sign.^b^ | No. of Sites with ω > 1^c^ |
| --- | --- | --- | --- | --- | --- |
|  |  |  |  |  | **B.E.B** |
|  |  |  |  |  |  |
| M0 (One ratio) | -10497.348696 | 0.40 | = ω_0_ |  | - |
|  |  |  |  |  |  |
| M1 (Neutral) | -10232.585227 | 0.70 | P_0_: 0.356  ω_0_: 0.16  P_1_: 0.643  ω1: 1.0 | P << 0.001 | - |
| M2 (Selection)* | -10183.919621 | 1.03 | P_0_: 0.175  ω_0_: 0.02  P_1_: 0.648  ω_1_:1.0  P_2_: 0.175  ω_2_: 2.16 |  | 6 (PP ≥ 0.99)  2 (P > 0.95) |
| M3 (Discrete)* | -10094.820408 | 0.53 | P_0_: 0.174  ω_0_: 0.01  P_1_: 0.596  ω_1_: 0.44  P_2_: 0.228  ω_2_: 1.17 | P << 0.001 | - |
| M7 (beta) | -10100.519570 | 0.46 | p: 0.54825  q: 0.62311 | P << 0.001 | - |
| M8 (beta and ω)* | -10091.003742 | 0.53 | p_0_: 0.915  p: 0.607  q: 0.779  p1: 0.08  ω: 1.53 |  | 2 (PP ≥ 0.99)  2 (P > 0.95) |
|  |  |  |  |  |  |

**Legend:**

**a:** dn/ds (weighted average)

**b:** Significance of the model in comparison with the null model

**c:** Number of sites with ω > 1 under the Bayes empirical Bayes approach with a posterior probability (PP) more than or equal to 0.99 and 0.95

***** Models which allow ω > 1

**Table 2.5** Maximum-likelihood parameter estimates for long-K_V_-CSα/β

| Model | Likelihood () | ω_0_^a^ | Parameters | Sign.^b^ | No. of Sites with ω > 1^c^ |
| --- | --- | --- | --- | --- | --- |
|  |  |  |  |  | **B.E.B** |
|  |  |  |  |  |  |
| M0 (One ratio) | -6240.138995 | 0.26 | = ω_0_ |  | - |
|  |  |  |  |  |  |
| M1 (Neutral) | -6219.395807 | 0.54 | P_0_: 0.604  ω_0_: 0.24  P_1_: 0.395  ω1: 1.0 | P << 0.001 | - |
| M2 (Selection)* | -6290.393551 | 0.90 | P_0_: 0.097  ω_0_: 0.0  P_1_: 0.793  ω_1_:1.0  P_2_: 0.109  ω_2_: 1.0 |  | 0 (PP ≥ 0.99)  0 (P > 0.95) |
| M3 (Discrete)* | -6158.017172 | 0.28 | P_0_: 0.0001  ω_0_: 0.0  P_1_: 0.096  ω_1_: 0.0  P_2_: 0.902  ω_2_: 0.31 | P << 0.001 | - |
| M7 (beta) | -6144.555242 | 0.29 | p: 1.07220  q: 2.57764 | P > 0.05^NS^ | - |
| M8 (beta and ω)* | -6144.555803 | 0.29 | p_0_: 0.999  p: 1.07  q: 2.57  p1: 0.00001  ω: 1.0 |  | 0 (PP ≥ 0.99)  0 (P > 0.95) |
|  |  |  |  |  |  |

**Legend:**

**a:** dn/ds (weighted average)

**b:** Significance of the model in comparison with the null model

**c:** Number of sites with ω > 1 under the Bayes empirical Bayes approach with a posterior probability (PP) more than or equal to 0.99 and 0.95

***** Models which allow ω > 1

**P > 0.05^NS^:** Not significant at 0.05

**Table 2.6** Maximum-likelihood parameter estimates for short-K_V_-CSα/β

| Model | Likelihood () | ω_0_^a^ | Parameters | Sign.^b^ | No. of Sites with ω > 1^c^ |
| --- | --- | --- | --- | --- | --- |
|  |  |  |  |  | **B.E.B** |
|  |  |  |  |  |  |
| M0 (One ratio) | -9277.303143 | 0.34 | = ω_0_ |  | - |
|  |  |  |  |  |  |
| M1 (Neutral) | -9052.776032 | 0.88 | P_0_: 0.118  ω_0_: 0  P_1_: 0.881  ω_1_: 1.0 | P << 0.001 | - |
| M2 (Selection)* | -9028.103335 | 1.02 | P_0_: 0.118  ω_0_: 0  P_1_: 0.791  ω_1_:1.0  P_2_: 0.089  ω_2_: 2.55 |  | 3 (PP ≥ 0.99)  2 (P > 0.95) |
| M3 (Discrete)* | -8973.108747 | 0.38 | P_0_: 0.058  ω_0_: 0  P_1_: 0.059  ω_1_: 0  P_2_: 0.881  ω_2_: 0.43 | P << 0.001 | - |
| M7 (beta) | -8909.248640 | 0.39 | p: 0.50659  q: 0.77287 | P > 0.05^NS^ | - |
| M8 (beta and ω)* | -8908.269923 | 0.42 | p_0_: 0.963  p: 0.528  q: 0.860  p1: 0.036  ω: 1.51 |  | 0 (PP ≥ 0.99)  0 (P > 0.95) |
|  |  |  |  |  |  |

**Legend:**

**a:** dn/ds (weighted average)

**b:** Significance of the model in comparison with the null model

**c:** Number of sites with ω > 1 under the Bayes empirical Bayes approach with a posterior probability (PP) more than or equal to 0.99 and 0.95

***** Models which allow ω > 1

**P > 0.05^NS^:** Not significant at 0.05

**Table 2.7** Maximum-likelihood parameter estimates for Cl_V_-CSα/β

| Model | Likelihood () | ω_0_^a^ | Parameters | Sign.^b^ | No. of Sites with ω > 1^c^ |
| --- | --- | --- | --- | --- | --- |
|  |  |  |  |  | **B.E.B** |
|  |  |  |  |  |  |
| M0 (One ratio) | -1395.129859 | 0.40 | = ω_0_ |  | - |
|  |  |  |  |  |  |
| M1 (Neutral) | -1336.586472 | 0.49 | P_0_: 0.540  ω_0_: 0.07  P_1_: 0.459  ω1: 1.0 | P << 0.001 | - |
| M2 (Selection)* | -1335.489235 | 0.62 | P_0_: 0.537  ω_0_: 0.08  P_1_: 0.307  ω_1_: 1.0  P_2_: 0.154  ω_2_: 1.78 |  | 0 (PP ≥ 0.99)  0 (P > 0.95) |
| M3 (Discrete)* | -1335.478349 | 0.62 | P_0_: 0.535  ω_0_: 0.08  P_1_: 0.282  ω_1_: 0.94  P_2_: 0.181  ω_2_: 1.70 | P > 0.05^NS^ | - |
| M7 (beta) | -1346.048427 | 0.44 | p: 0.37688  q: 0.47585 | P << 0.001 | - |
| M8 (beta and ω)* | -1336.103180 | 0.60 | p_0_: 0.552  p: 9.692  q: 99.0  p_1_: 0.447  ω: 1.23 |  | 0 (PP ≥ 0.99)  2 (P > 0.95) |
|  |  |  |  |  |  |

**Legend:**

**a:** dn/ds (weighted average)

**b:** Significance of the model in comparison with the null model

**c:** Number of sites with ω > 1 under the Bayes empirical Bayes approach with a posterior probability (PP) more than or equal to 0.99 and 0.95

***** Models which allow ω > 1

**P > 0.05^NS^:** Not significant at 0.05
