# Supplementary material for: Evolution Stings: The Origin and Diversification of Scorpion Toxin Peptide Scaffolds
Source: Toxins (Basel). 2013 Dec 13;5(12):2456–87. doi: 10.3390/toxins5122456 (PMC3873696; doi:10.3390/toxins5122456)
Supplement: Supplementary File 1 — Supplementary (ZIP, 4932 KB) [file toxins-05-02456-s001.zip › Supplementary Table 3.1-3.8 - Selection analyses details for non-CSab toxins.docx]

**Table 3.1** Maximum-likelihood parameter estimates for the SV-SVCs

| Model | Likelihood () | ω_0_^a^ | Parameters | Sign.^b^ | No. of Sites with ω > 1^c^ |
| --- | --- | --- | --- | --- | --- |
|  |  |  |  |  | **B.E.B** |
|  |  |  |  |  |  |
| M0 (One ratio) | -4504.672697 | 0.25 | = ω_0_ |  | - |
|  |  |  |  |  |  |
| M1 (Neutral) | -4464.933621 | 0.68 | P_0_: 0.393  ω_0_: 0.19  P_1_: 0.606  ω1:1.0 | P > 0.05^NS^ | - |
| M2 (Selection)* | -4464.933621 | 0.68 | P_0_: 0.393  ω_0_: 0.19  P_1_: 0.463  ω_1_:1.0  P_2_: 0.143  ω_2_: 1.0 |  | 0 (PP ≥ 0.99)  0 (P ≥ 0.95) |
| M3 (Discrete)* | -4390.634714 | 0.31 | P_0_: 0.089  ω_0_: 0.001  P_1_: 0.471  ω_1_: 0.19  P_2_: 0.439  ω_2_: 0.49 | P << 0.001 | - |
| M7 (beta) | -4407.098727 | 0.34 | p: 0.76324  q: 1.43231 | P > 0.05^NS^ | - |
| M8 (beta and ω)* | -4407.098847 | 0.34 | p_0_: 0.999  p: 0.763  q: 1.43  p1: 0.00001  ω: 1.0 |  | 0 (PP ≥ 0.99)  0 (P ≥ 0.95) |
|  |  |  |  |  |  |

**Legend:**

**a:** dn/ds (weighted average)

**b:** Significance of the model in comparison with the null model

**c:** Number of sites with ω > 1 under the Bayes empirical Bayes approach with a posterior probability (PP) more than or equal to 0.99 and 0.95

***** Models which allow ω > 1

**P > 0.05^NS^:** Not significant at 0.05

**Table 3.2** Maximum-likelihood parameter estimates for ICK

| Model | Likelihood () | ω_0_^a^ | Parameters | Sign.^b^ | No. of Sites with ω > 1^c^ |
| --- | --- | --- | --- | --- | --- |
|  |  |  |  |  | **B.E.B** |
|  |  |  |  |  |  |
| M0 (One ratio) | -1136.806829 | 0.26 | = ω_0_ |  | - |
|  |  |  |  |  |  |
| M1 (Neutral) | -1126.688551 | 0.49 | P_0_: 0.611  ω_0_: 0.17  P_1_: 0.388  ω1: 1.0 | P > 0.05^NS^ | - |
| M2 (Selection)* | -1126.688551 | 0.49 | P_0_: 0.611  ω_0_: 0.17  P_1_: 0.386  ω_1_:1.0  P_2_: 0.001  ω_2_: 1.0 |  | 0 (PP ≥ 0.99)  0 (P ≥ 0.95) |
| M3 (Discrete)* | -1117.257283 | 0.32 | P_0_: 0.231  ω_0_: 0.01  P_1_: 0.631  ω_1_: 0.32  P_2_: 0.137  ω_2_: 0.85 | P << 0.001 | - |
| M7 (beta) | -1118.986407 | 0.34 | p: 0.69454  q: 1.31516 | P > 0.05^NS^ | - |
| M8 (beta and ω)* | -1118.986438 | 0.34 | p_0_: 0.999  p: 0.694  q: 1.31  p1: 0.00001  ω: 1.0 |  | 0 (PP ≥ 0.99)  0 (P ≥ 0.95) |
|  |  |  |  |  |  |

**Legend:**

**a:** dn/ds (weighted average)

**b:** Significance of the model in comparison with the null model

**c:** Number of sites with ω > 1 under the Bayes empirical Bayes approach with a posterior probability (PP) more than or equal to 0.99 and 0.95

***** Models which allow ω > 1

**P > 0.05^NS^:** Not significant at 0.05

**Table 3.3** Maximum-likelihood parameter estimates for DDH

| Model | Likelihood () | ω_0_^a^ | Parameters | Sign.^b^ | No. of Sites with ω > 1^c^ |
| --- | --- | --- | --- | --- | --- |
|  |  |  |  |  | **B.E.B** |
|  |  |  |  |  |  |
| M0 (One ratio) | -757.331431 | 0.32 | = ω_0_ |  | - |
|  |  |  |  |  |  |
| M1 (Neutral) | -757.331545 | 0.32 | P_0_: 0.999  ω_0_: 0.32  P_1_: 0.00001  ω_1_: 1.0 | P > 0.05^NS^ | - |
| M2 (Selection)* | -757.331431 | 0.32 | P_0_: 1.0  ω_0_: 0.32  P_1_: 0  ω_1_:1.0  P_2_: 0  ω_2_: 1.0 |  | 0 (PP ≥ 0.99)  0 (P ≥ 0.95) |
| M3 (Discrete)* | -757.331431 | 0.32 | P_0_: 0.188  ω_0_: 0.32  P_1_: 0.361  ω_1_: 0.32  P_2_: 0.449  ω_2_: 0.32 | P > 0.05^NS^ | - |
| M7 (beta) | -757.354870 | 0.32 | p: 47.86273  q: 99.0 | P > 0.05^NS^ | - |
| M8 (beta and ω)* | -757.354983 | 0.32 | p_0_: 0.999  p: 47.86  q: 99.0  p1: 0.00001  ω: 1.0 |  | 0 (PP ≥ 0.99)  0 (P ≥ 0.95) |
|  |  |  |  |  |  |

**Legend:**

**a:** dn/ds (weighted average)

**b:** Significance of the model in comparison with the null model

**c:** Number of sites with ω > 1 under the Bayes empirical Bayes approach with a posterior probability (PP) more than or equal to 0.99 and 0.95

***** Models which allow ω > 1

**P > 0.05^NS^:** Not significant at 0.05

**Table 3.4** Maximum-likelihood parameter estimates for AMP

| Model | Likelihood () | ω_0_^a^ | Parameters | Sign.^b^ | No. of Sites with ω > 1^c^ |
| --- | --- | --- | --- | --- | --- |
|  |  |  |  |  | **B.E.B** |
|  |  |  |  |  |  |
| M0 (One ratio) | -3576.689959 | 0.30 | = ω_0_ |  | - |
|  |  |  |  |  |  |
| M1 (Neutral) | -3561.915610 | 0.34 | P_0_: 0.906  ω_0_: 0.27  P_1_: 0.093  ω1: 1.0 | P > 0.05^NS^ | - |
| M2 (Selection)* | -3561.915610 | 0.34 | P_0_: 0.906  ω_0_: 0.27  P_1_: 0.044  ω_1_:1.0  P_2_: 0.048  ω_2_: 1.0 |  | 0 (PP ≥ 0.99)  0 (P ≥ 0.95) |
| M3 (Discrete)* | -3547.008218 | 0.33 | P_0_: 0.256  ω_0_: 0.11  P_1_: 0.716  ω_1_: 0.36  P_2_: 0.027  ω_2_: 1.82 | P << 0.001 | - |
| M7 (beta) | -3549.603412 | 0.32 | p: 1.94058  q: 4.08835 | P > 0.05^NS^ | - |
| M8 (beta and ω)* | -3546.114180 | 0.33 | p_0_: 0.977  p: 2.411  q: 5.54  p_1_: 0.022  ω: 1.88 |  | 0 (PP ≥ 0.99)  0 (P ≥ 0.95) |
|  |  |  |  |  |  |

**Legend:**

**a:** dn/ds (weighted average)

**b:** Significance of the model in comparison with the null model

**c:** Number of sites with ω > 1 under the Bayes empirical Bayes approach with a posterior probability (PP) more than or equal to 0.99 and 0.95

***** Models which allow ω > 1

**P > 0.05^NS^:** Not significant at 0.05

**Table 3.5** Maximum-likelihood parameter estimates for linear toxins

| Model | Likelihood () | ω_0_^a^ | Parameters | Sign.^b^ | No. of Sites with ω > 1^c^ |
| --- | --- | --- | --- | --- | --- |
|  |  |  |  |  | **B.E.B** |
|  |  |  |  |  |  |
| M0 (One ratio) | -4391.151429 | 0.24 | = ω_0_ |  | - |
|  |  |  |  |  |  |
| M1 (Neutral) | -4352.845142 | 0.42 | P_0_: 0.733  ω_0_: 0.21  P_1_: 0.266  ω1: 1.0 | P > 0.05^NS^ | - |
| M2 (Selection)* | -4352.845142 | 0.42 | P_0_: 0.733  ω_0_: 0.210  P_1_: 0.158  ω_1_:1.0  P_2_: 0.107  ω_2_: 1.0 |  | 0 (PP ≥ 0.99)  0 (P ≥ 0.95) |
| M3 (Discrete)* | -4313.670086 | 0.28 | P_0_: 0.302  ω_0_: 0.06  P_1_: 0.467  ω_1_: 0.26  P_2_: 0.229  ω_2_: 0.60 | P << 0.001 | - |
| M7 (beta) | -4312.174226 | 0.27 | p: 1.08497  q: 2.76298 | P > 0.05^NS^ | - |
| M8 (beta and ω)* | -4312.174906 | 0.27 | p_0_: 0.999  p: 1.08  q: 2.76  p_1_: 0.00001  ω: 8.58 |  | 0 (PP ≥ 0.99)  0 (P ≥ 0.95) |
|  |  |  |  |  |  |

**Legend:**

**a:** dn/ds (weighted average)

**b:** Significance of the model in comparison with the null model

**c:** Number of sites with ω > 1 under the Bayes empirical Bayes approach with a posterior probability (PP) more than or equal to 0.99 and 0.95

***** Models which allow ω > 1

**P > 0.05^NS^:** Not significant at 0.05

**Table 3.6** Maximum-likelihood parameter estimates for short-chain Bradykinins

| Model | Likelihood () | ω_0_^a^ | Parameters | Sign.^b^ | No. of Sites with ω > 1^c^ |
| --- | --- | --- | --- | --- | --- |
|  |  |  |  |  | **B.E.B** |
|  |  |  |  |  |  |
| M0 (One ratio) | -2243.274812 | 0.18 | = ω_0_ |  | - |
|  |  |  |  |  |  |
| M1 (Neutral) | -2240.097369 | 0.22 | P_0_: 0.936  ω_0_: 0.17  P_1_: 0.063  ω_1_: 1.0 | P > 0.05^NS^ | - |
| M2 (Selection)* | -2240.097369 | 0.22 | P_0_: 0.936  ω_0_: 0.17  P_1_: 0.017  ω_1_:1.0  P_2_: 0.045  ω_2_: 1.0 |  | 0 (PP ≥ 0.99)  0 (P ≥ 0.95) |
| M3 (Discrete)* | -2227.326070 | 0.19 | P_0_: 0.023  ω_0_: 0.0  P_1_: 0.456  ω_1_: 0.09  P_2_: 0.520  ω_2_: 0.29 | P << 0.001 | - |
| M7 (beta) | -2228.289137 | 0.20 | p: 2.02643  q: 7.93356 | P > 0.05^NS^ | - |
| M8 (beta and ω)* | -2228.265033 | 0.20 | p_0_: 0.996  p: 2.06  q: 8.22  p1: 0.003  ω: 2.20 |  | 0 (PP ≥ 0.99)  0 (P ≥ 0.95) |
|  |  |  |  |  |  |

**Legend:**

**a:** dn/ds (weighted average)

**b:** Significance of the model in comparison with the null model

**c:** Number of sites with ω > 1 under the Bayes empirical Bayes approach with a posterior probability (PP) more than or equal to 0.99 and 0.95

***** Models which allow ω > 1

**P > 0.05^NS^:** Not significant at 0.05

**Table 3.7** Maximum-likelihood parameter estimates for Anionic toxins

| Model | Likelihood () | ω_0_^a^ | Parameters | Sign.^b^ | No. of Sites with ω > 1^c^ |
| --- | --- | --- | --- | --- | --- |
|  |  |  |  |  | **B.E.B** |
|  |  |  |  |  |  |
| M0 (One ratio) | -1683.601176 | 0.17 | = ω_0_ |  | - |
|  |  |  |  |  |  |
| M1 (Neutral) | -1670.207689 | 0.27 | P_0_: 0.837  ω_0_: 0.13  P_1_: 0.162  ω1: 1.0 | P > 0.05^NS^ | - |
| M2 (Selection)* | -1670.207689 | 0.27 | P_0_: 0.837  ω_0_: 0.13  P_1_: 0.10  ω_1_: 1.0  P_2_: 0.06  ω_2_: 1.0 |  | 0 (PP ≥ 0.99)  0 (P ≥ 0.95) |
| M3 (Discrete)* | -1665.472501 | 0.20 | P_0_: 0.075  ω_0_: 0.0  P_1_: 0.706  ω_1_: 0.12  P_2_: 0.217  ω_2_: 0.55 | P << 0.001 | - |
| M7 (beta) | -1666.569572 | 0.19 | p: 1.14544  q: 4.58407 | P > 0.05^NS^ | - |
| M8 (beta and ω)* | -1665.613499 | 0.22 | p_0_: 0.930  p: 1.57  q: 8.0  p_1_: 0.069  ω: 1.0 |  | 0 (PP ≥ 0.99)  0 (P ≥ 0.95) |
|  |  |  |  |  |  |

**Legend:**

**a:** dn/ds (weighted average)

**b:** Significance of the model in comparison with the null model

**c:** Number of sites with ω > 1 under the Bayes empirical Bayes approach with a posterior probability (PP) more than or equal to 0.99 and 0.95

***** Models which allow ω > 1

**P > 0.05^NS^:** Not significant at 0.05

**Table 3.8** Maximum-likelihood parameter estimates for Glycine-rich toxins

| Model | Likelihood () | ω_0_^a^ | Parameters | Sign.^b^ | No. of Sites with ω > 1^c^ |
| --- | --- | --- | --- | --- | --- |
|  |  |  |  |  | **B.E.B** |
|  |  |  |  |  |  |
| M0 (One ratio) | -1556.811902 | 0.06 | = ω_0_ |  | - |
|  |  |  |  |  |  |
| M1 (Neutral) | -1538.049328 | 0.35 | P_0_: 0.698  ω_0_: 0.07  P_1_: 0.301  ω1: 1.0 | P > 0.05^NS^ | - |
| M2 (Selection)* | -1538.049328 | 0.35 | P_0_: 0.698  ω_0_: 0.07  P_1_: 0.060  ω_1_: 1.0  P_2_: 0.241  ω_2_: 1.0 |  | 0 (PP ≥ 0.99)  0 (P ≥ 0.95) |
| M3 (Discrete)* | -1522.673708 | 0.13 | P_0_: 0.284  ω_0_: 0.0  P_1_: 0.378  ω_1_: 0.07  P_2_: 0.336  ω_2_: 0.30 | P << 0.001 | - |
| M7 (beta) | -1524.034546 | 0.14 | p: 0.45798  q: 2.56423 | P > 0.05^NS^ | - |
| M8 (beta and ω)* | -1524.034619 | 0.14 | p_0_: 0.999  p: 0.457  q: 2.56  p1: 0.00001  ω: 1.0 |  | 0 (PP ≥ 0.99)  0 (P ≥ 0.95) |
|  |  |  |  |  |  |

**Legend:**

**a:** dn/ds (weighted average)

**b:** Significance of the model in comparison with the null model

**c:** Number of sites with ω > 1 under the Bayes empirical Bayes approach with a posterior probability (PP) more than or equal to 0.99 and 0.95

***** Models which allow ω > 1

**P > 0.05^NS^:** Not significant at 0.05
