# Supplementary figures and images for: Evolution Stings: The Origin and Diversification of Scorpion Toxin Peptide Scaffolds
Source: Toxins (Basel). 2013 Dec 13;5(12):2456–87. doi: 10.3390/toxins5122456 (PMC3873696; doi:10.3390/toxins5122456)

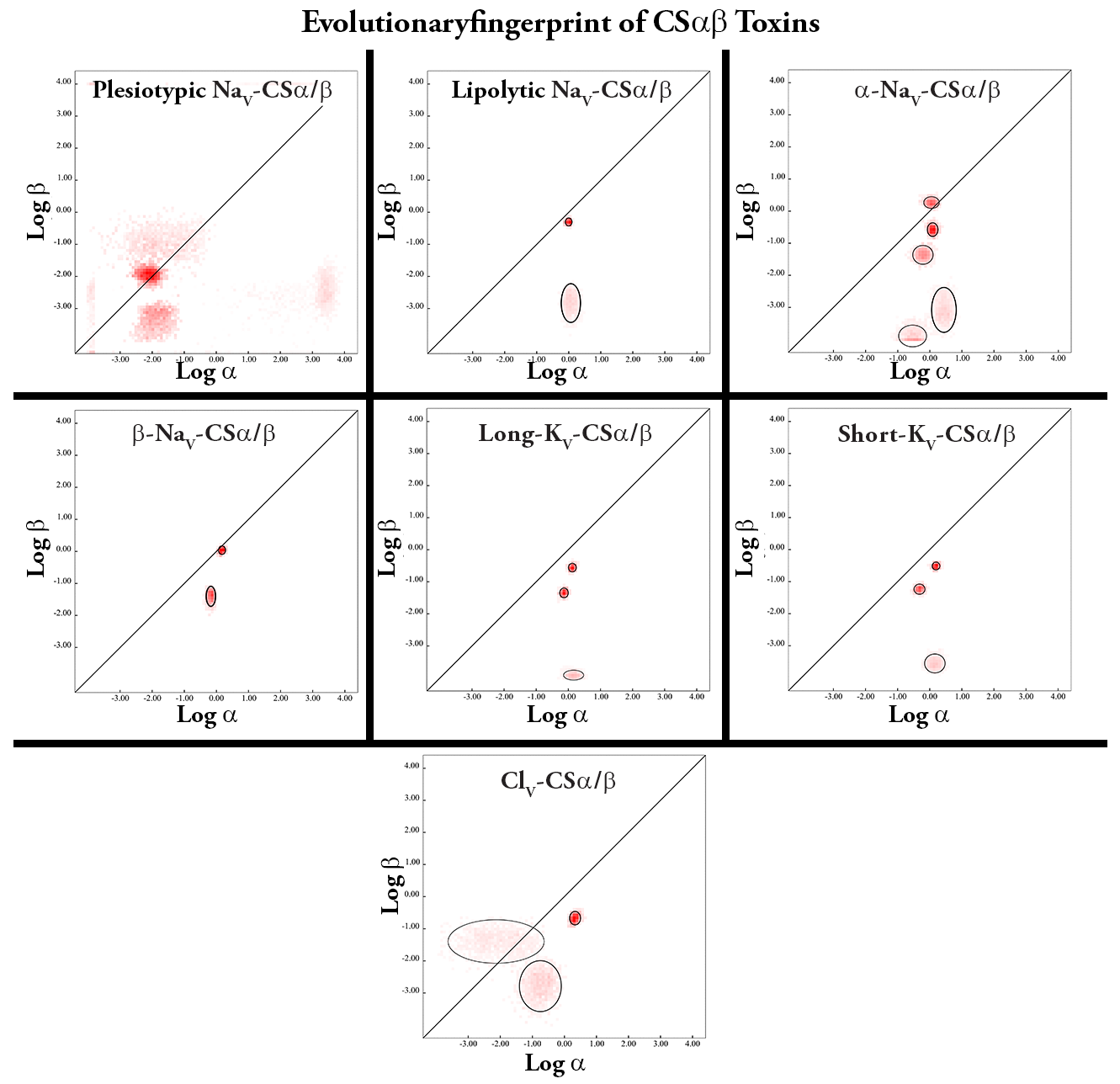

Supplement: Supplementary File 1 — Supplementary (ZIP, 4932 KB) [file toxins-05-02456-s001.zip › Supplementary Figure 1 - Evolutionaryfingerprint of Scorpion CSab toxins.tif]

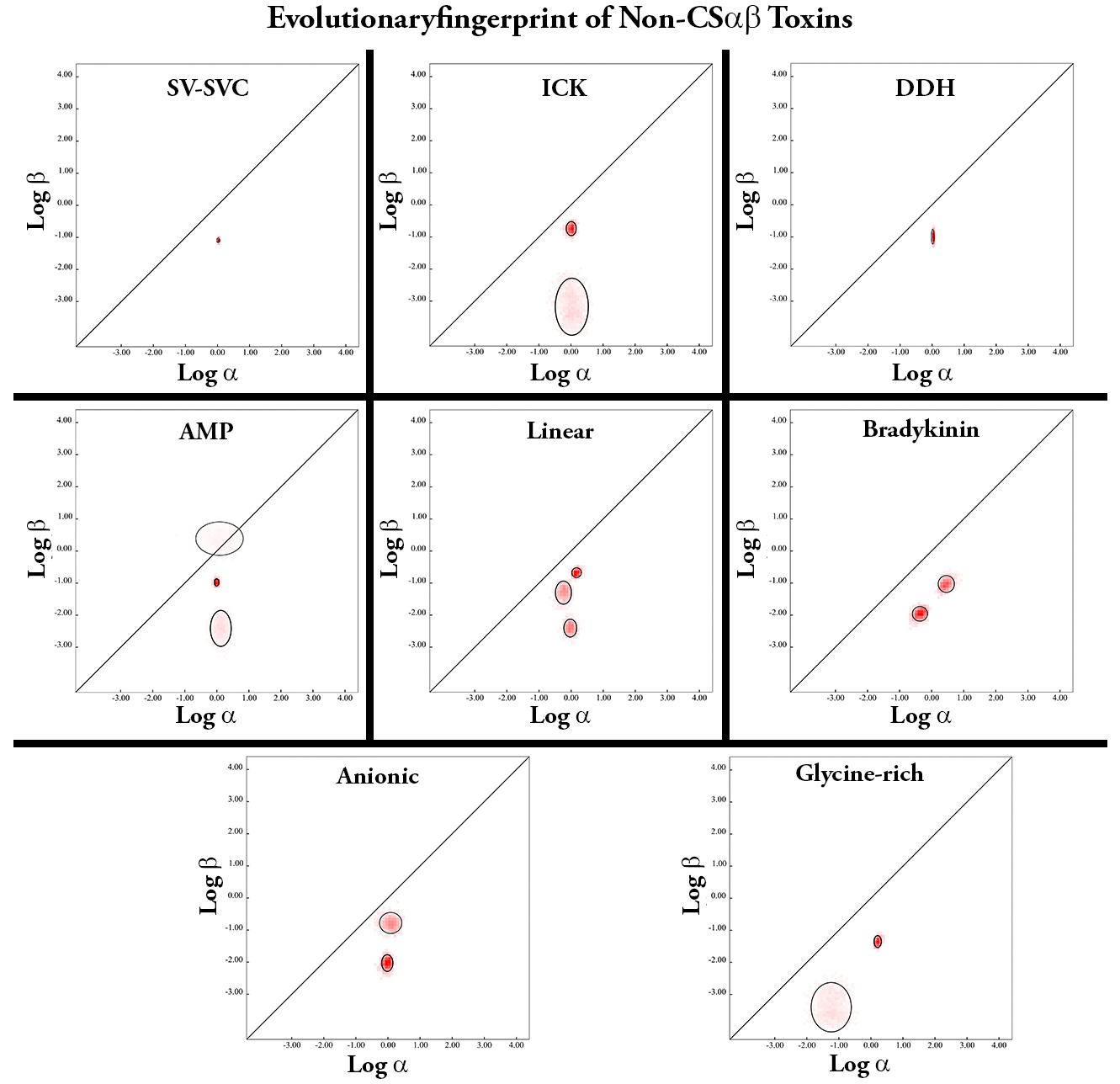

Supplement: Supplementary File 1 — Supplementary (ZIP, 4932 KB) [file toxins-05-02456-s001.zip › Supplementary Figure 2 - Evolutionaryfingerprint of Scorpion Non-CSab toxins.tif]

# $\alpha$ Na<sub>v</sub> CS $\alpha/\beta$

# $\beta$ Na<sub>v</sub> CS $\alpha/\beta$

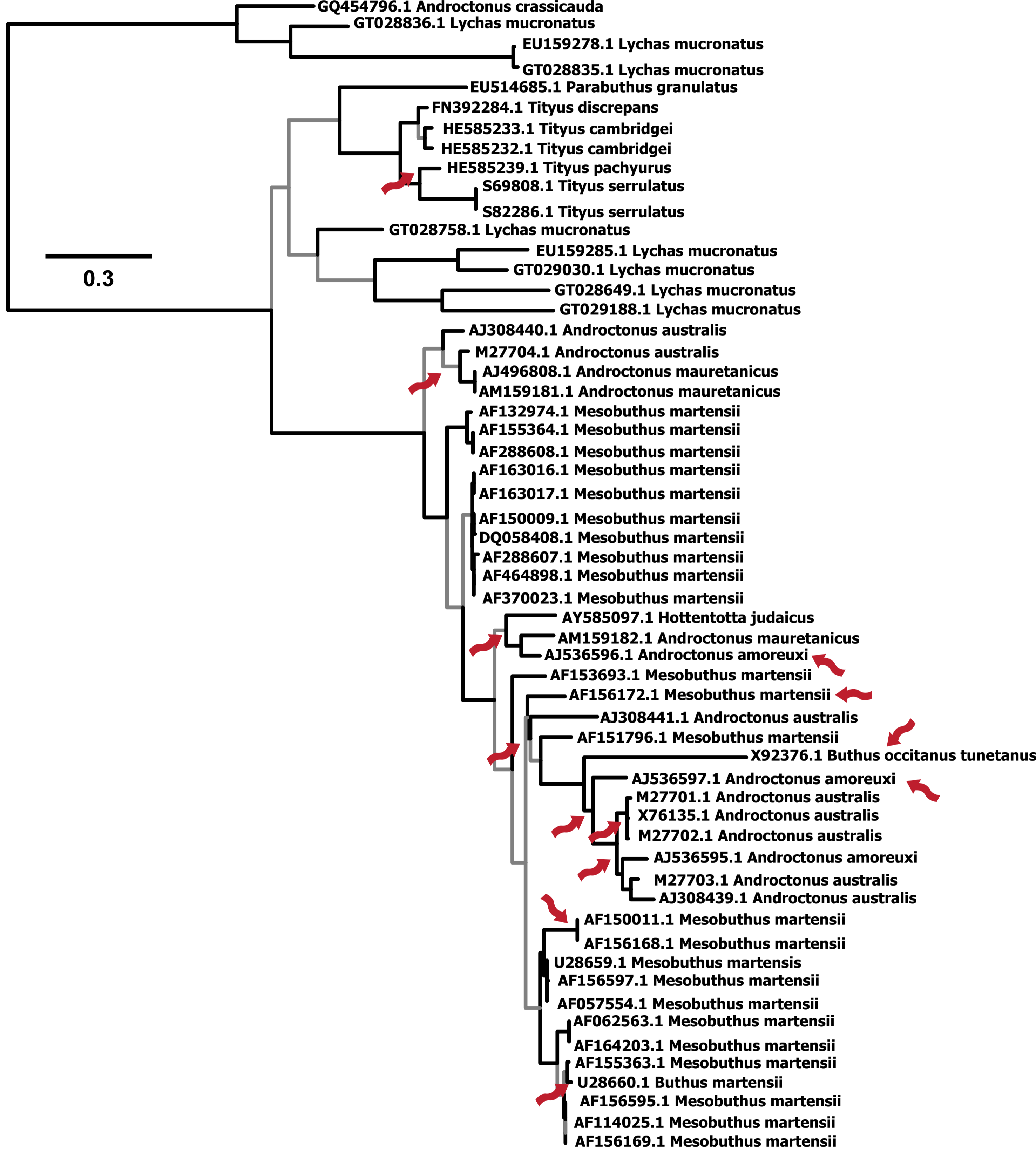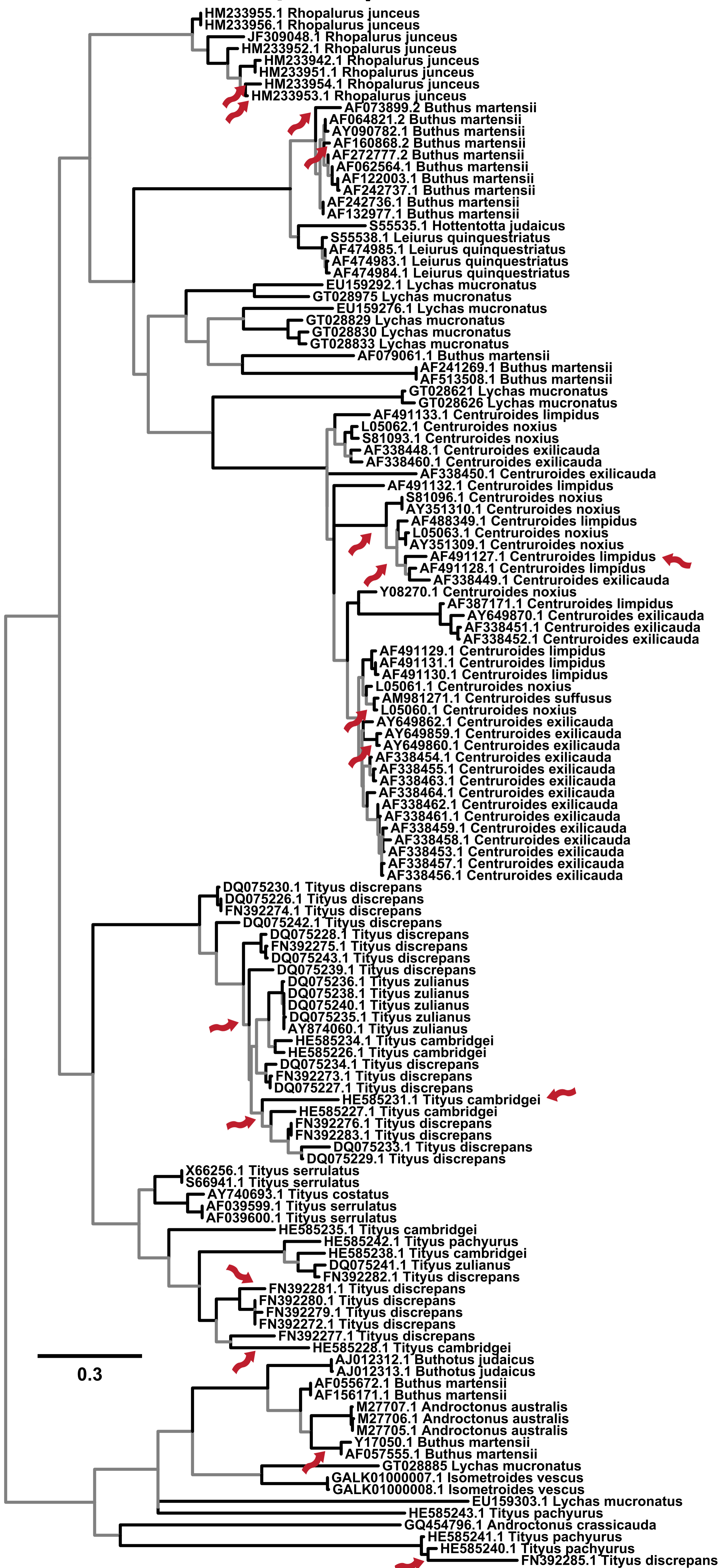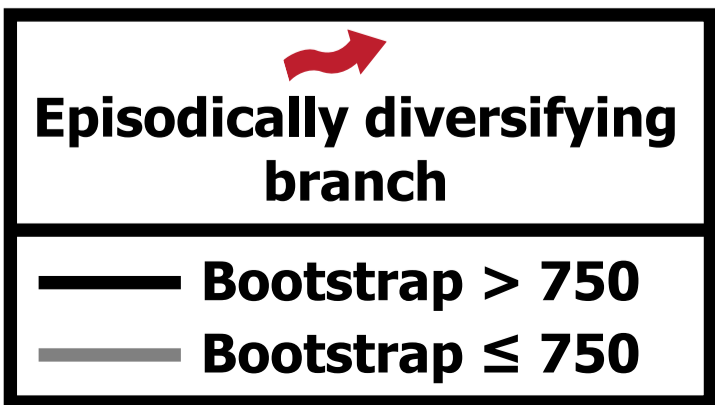

Supplement: Supplementary File 1 — Supplementary (ZIP, 4932 KB) [file toxins-05-02456-s001.zip › Supplementary Figure 4 - Alpha and Beta NaTxs.pdf]

Short K<sub>v</sub> CS<sub>α</sub>/β

Long K<sub>v</sub> CS<sub>α</sub>/β

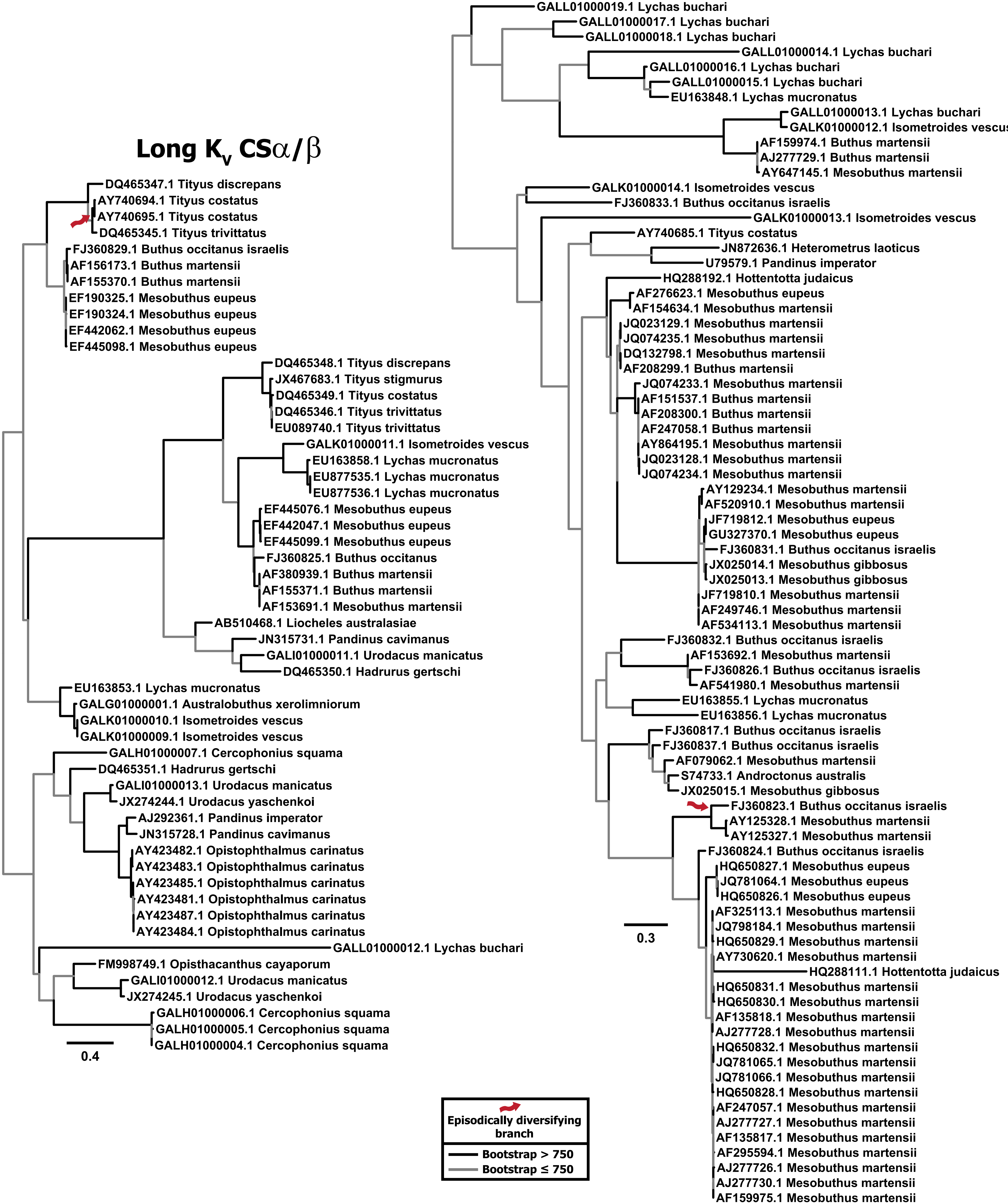

Supplement: Supplementary File 1 — Supplementary (ZIP, 4932 KB) [file toxins-05-02456-s001.zip › Supplementary Figure 5 - Long and Short KTxs.pdf]

# Anionic

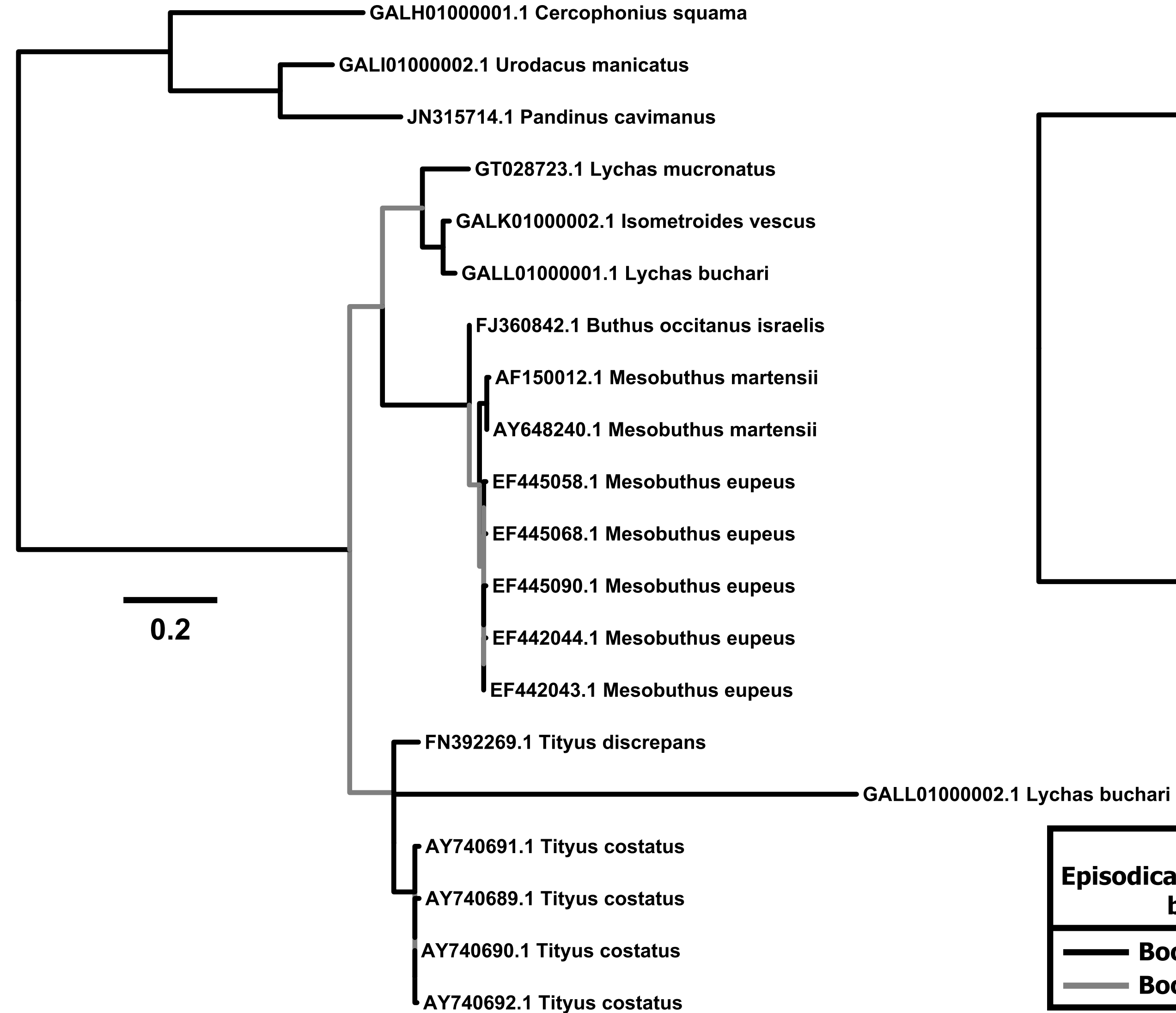

# Glycine-rich

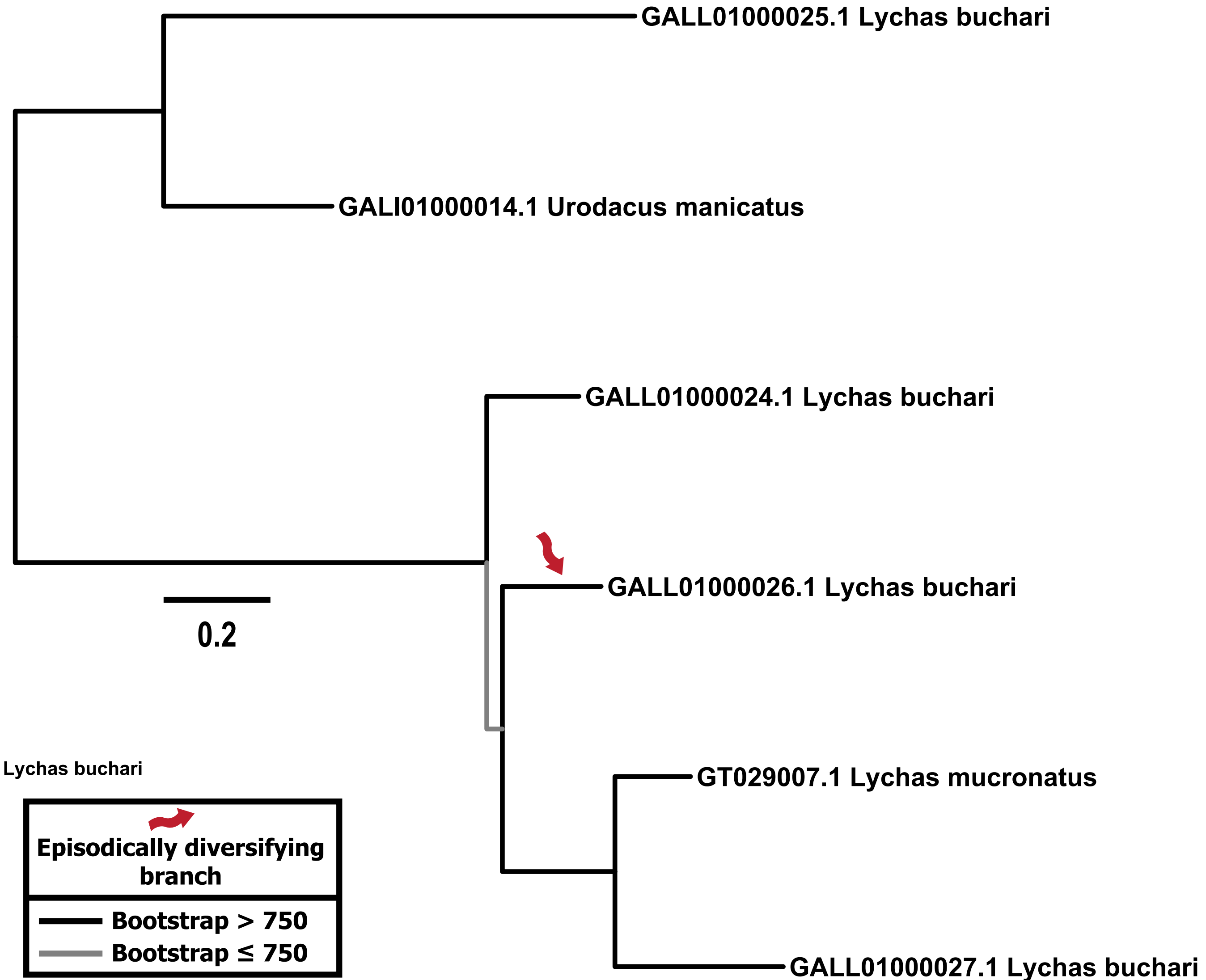

Supplement: Supplementary File 1 — Supplementary (ZIP, 4932 KB) [file toxins-05-02456-s001.zip › Supplementary Figure 7 - Glycine-rich and Anionic.pdf]

CYLIPs

AMP

Linear

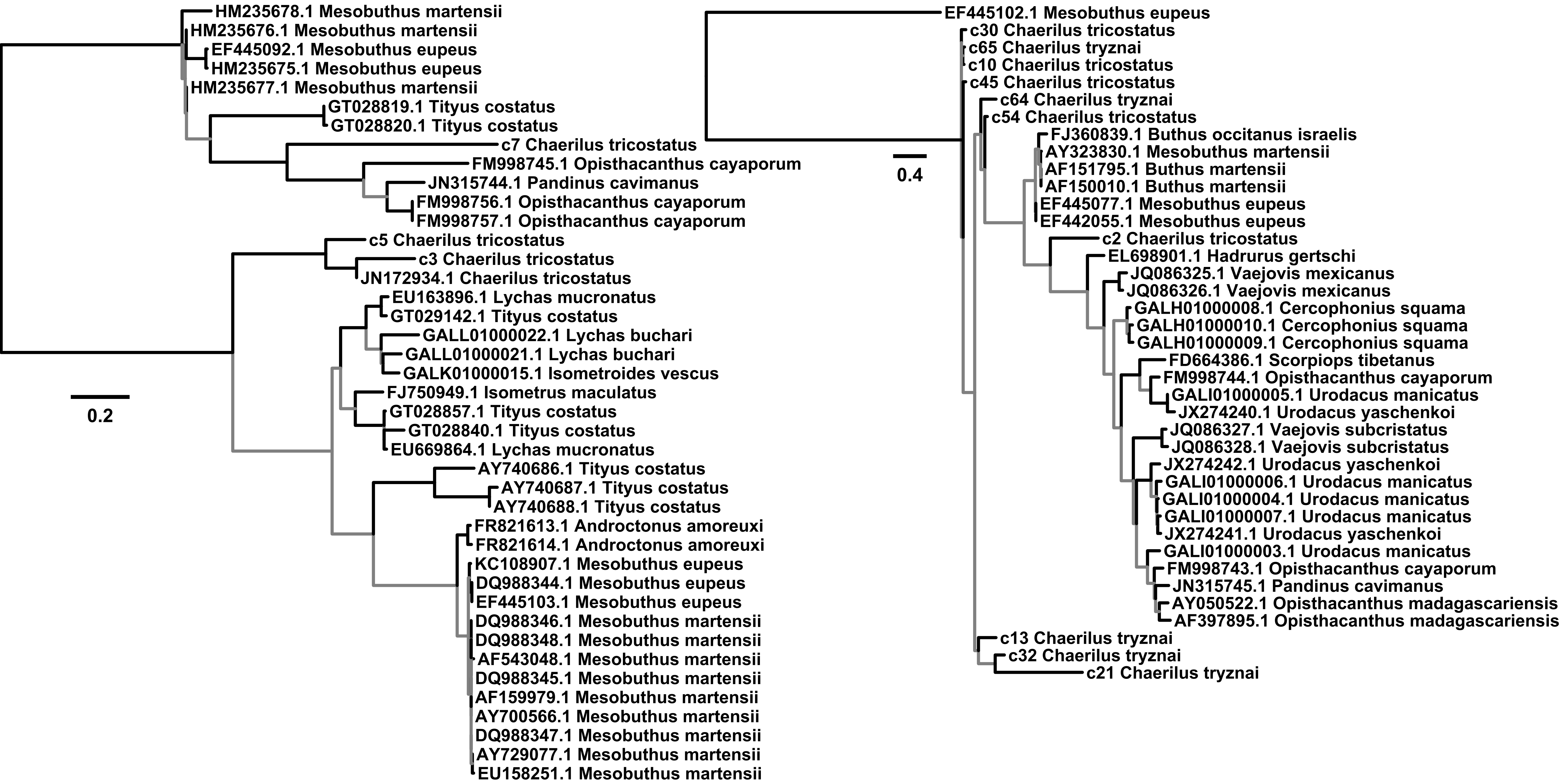

BPP

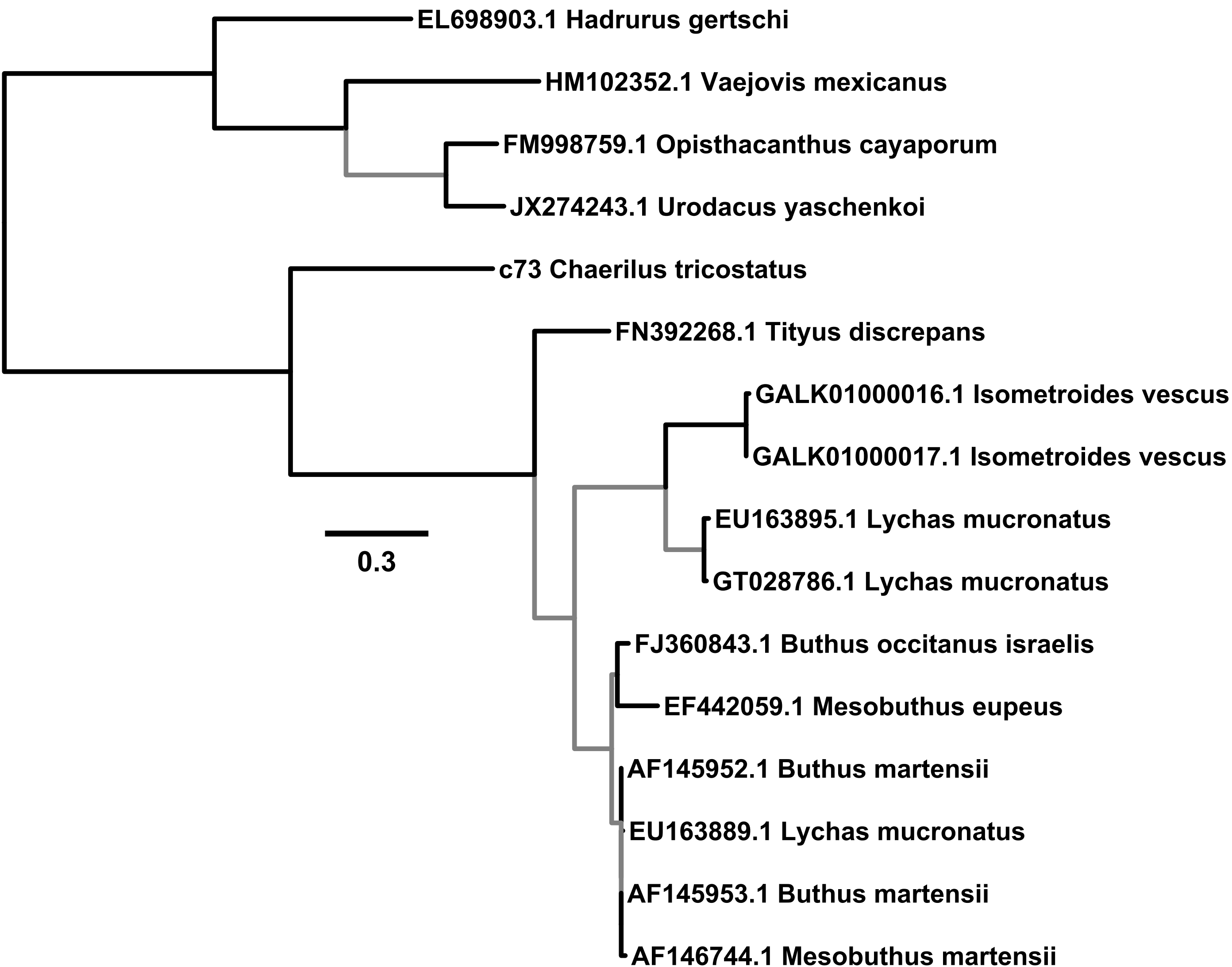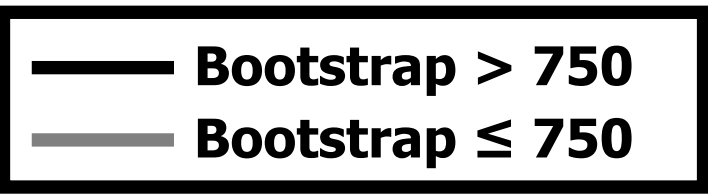

Supplement: Supplementary File 1 — Supplementary (ZIP, 4932 KB) [file toxins-05-02456-s001.zip › Supplementary Figure 8 - CYLIPs.pdf]

SV-SVC

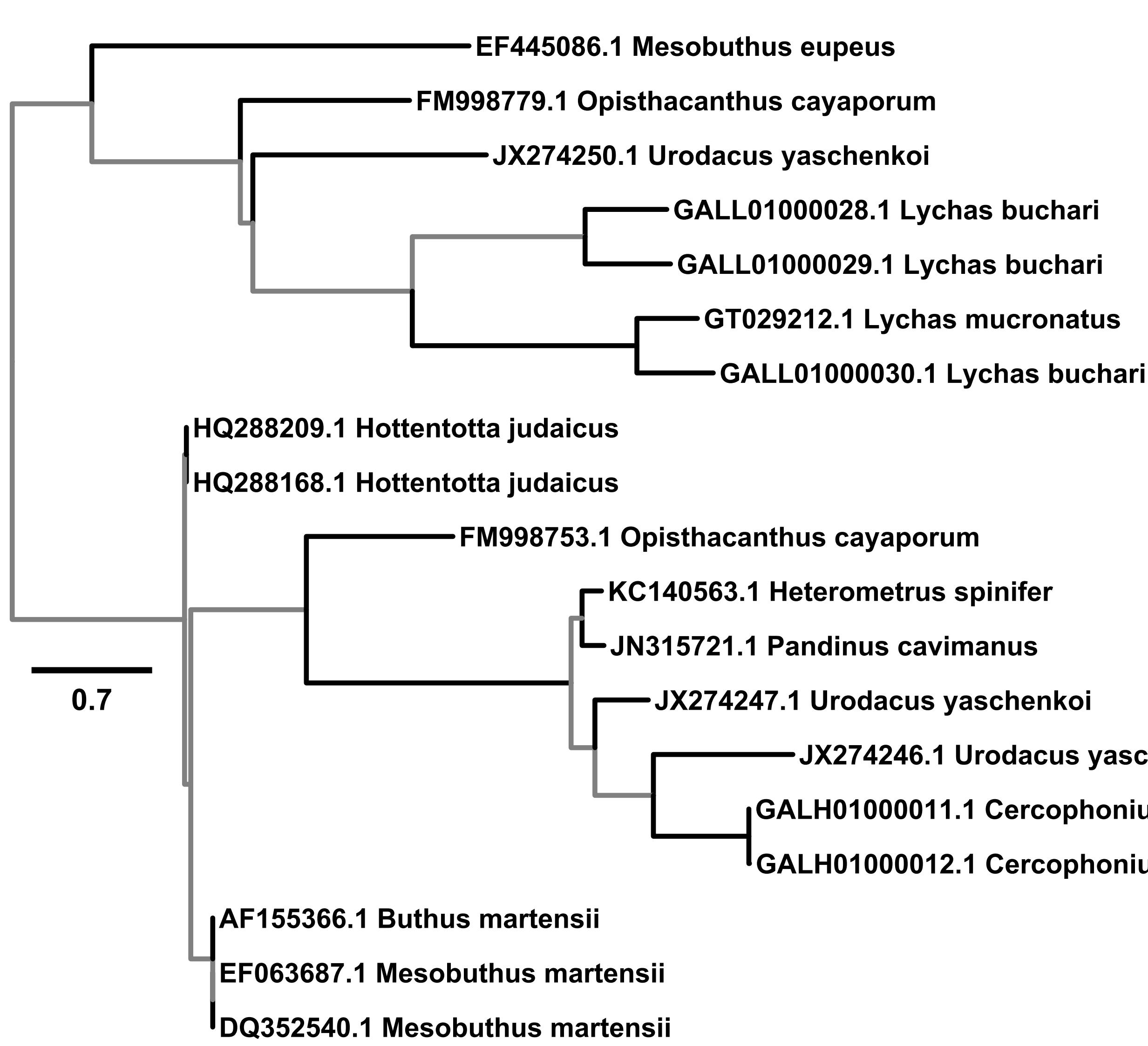

ICK

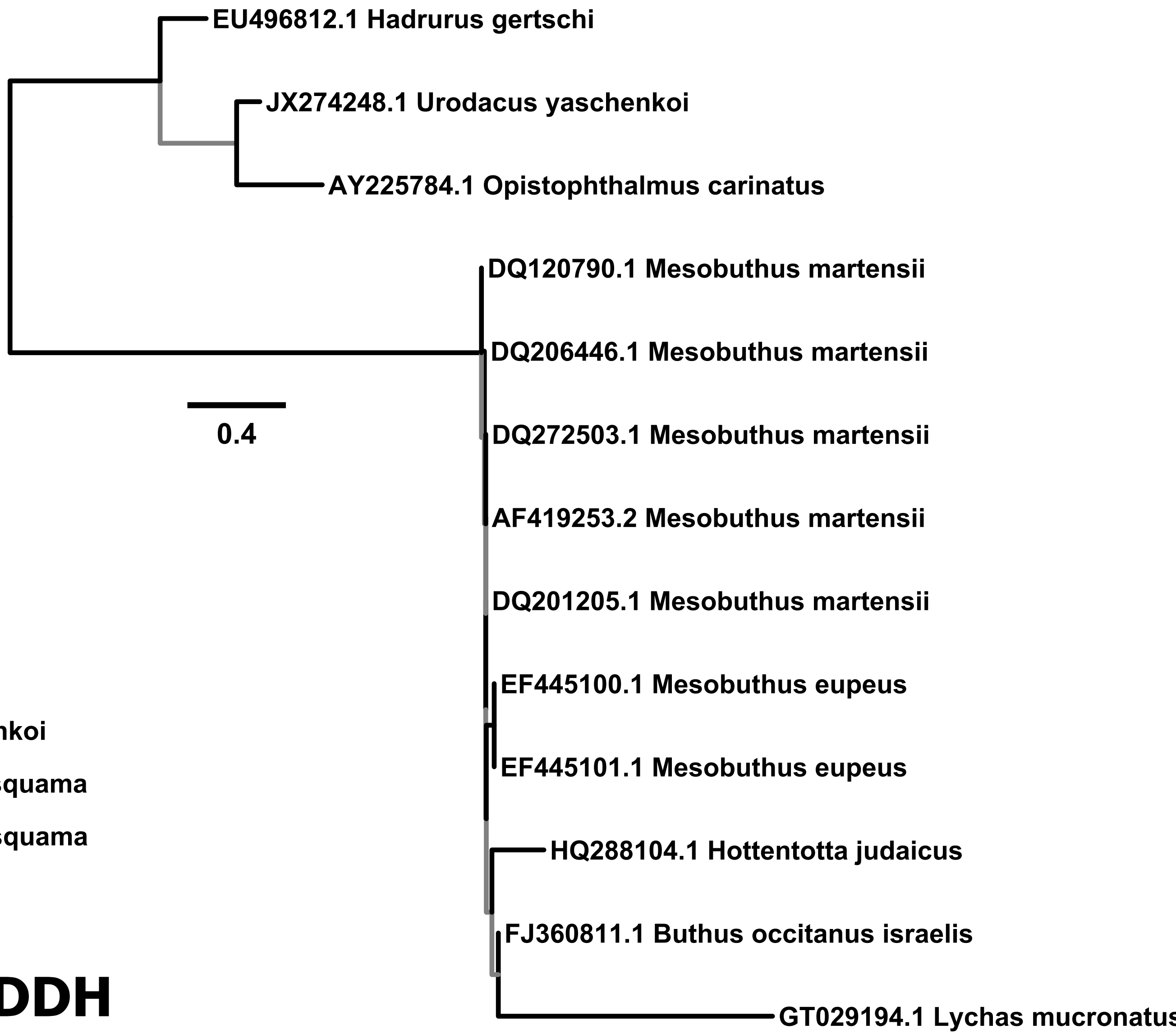

DDH

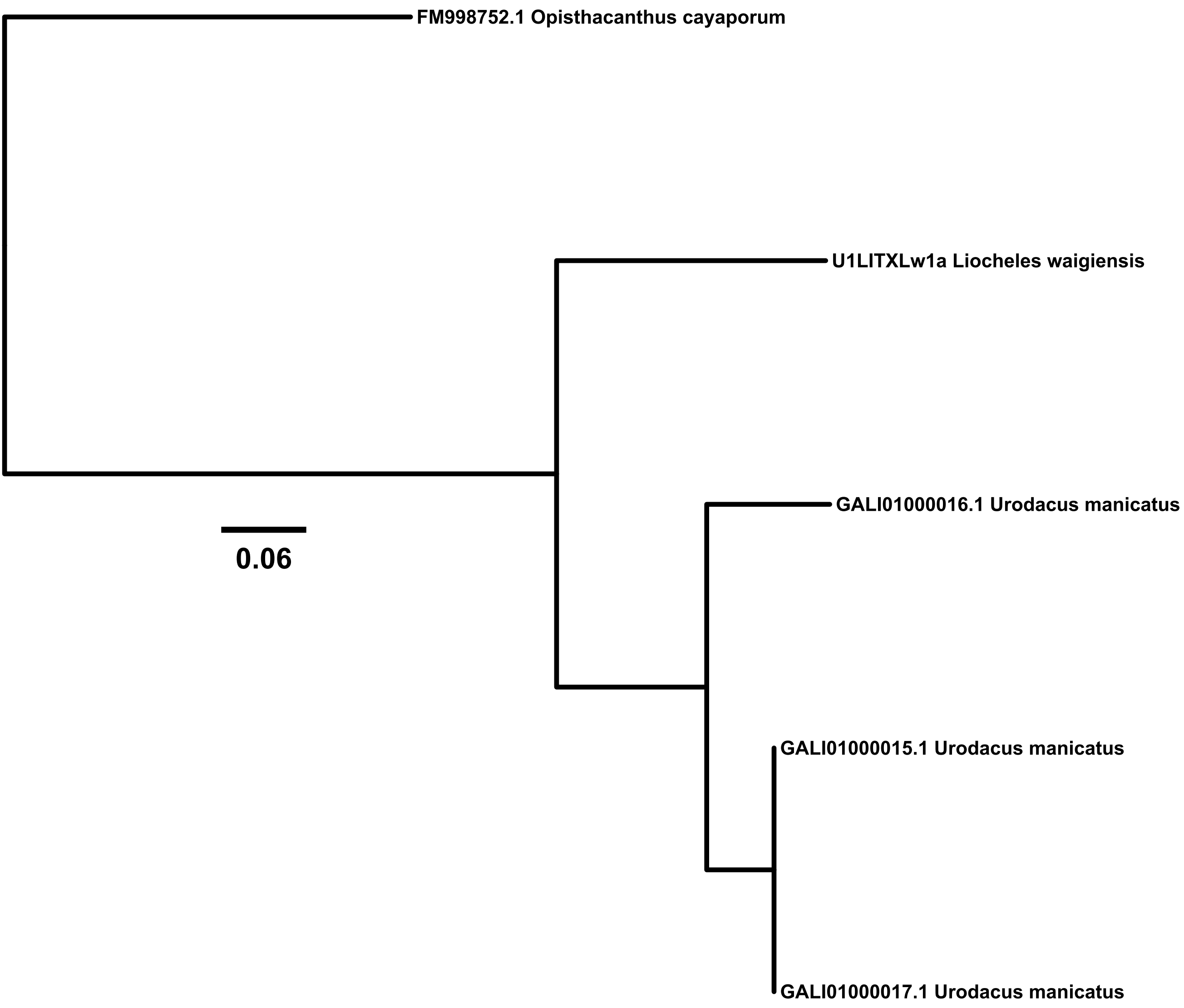

— Bootstrap > 750  
— Bootstrap ≤ 750

Supplement: Supplementary File 1 — Supplementary (ZIP, 4932 KB) [file toxins-05-02456-s001.zip › Supplementary Figure 9 - ICK DDH SV-SVC.pdf]
